# Supplementary figures and images for: Proteogenomic analyses indicate bacterial methylotrophy and archaeal heterotrophy are prevalent below the grass root zone
Source: PeerJ. 2016 Nov 8;4:e2687. doi: 10.7717/peerj.2687 (PMC5103831; doi:10.7717/peerj.2687)

**A**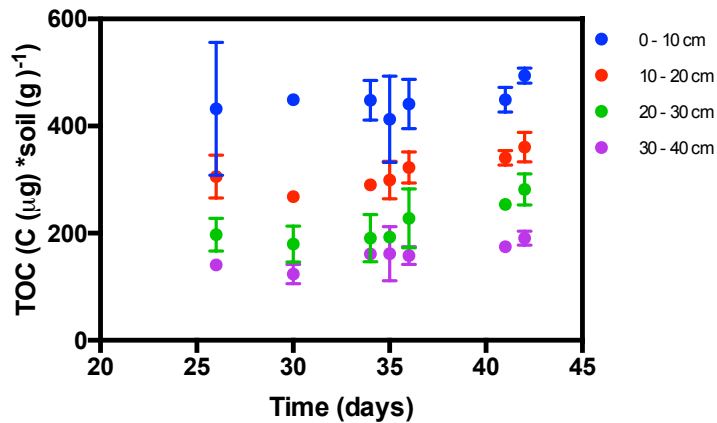**B**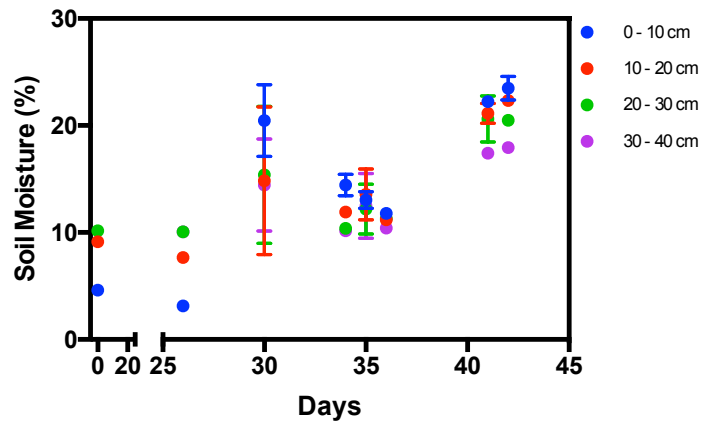**C**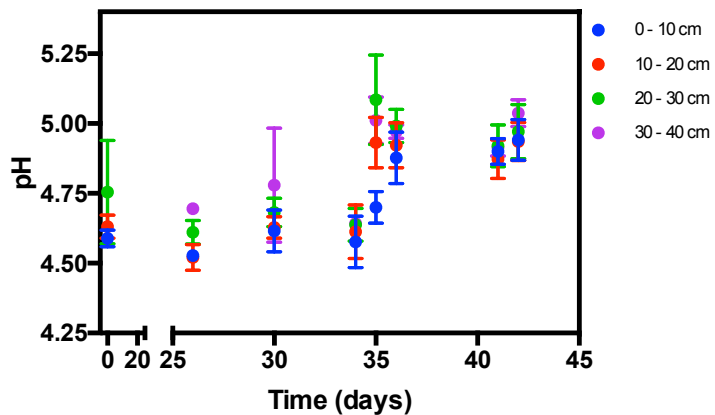**D**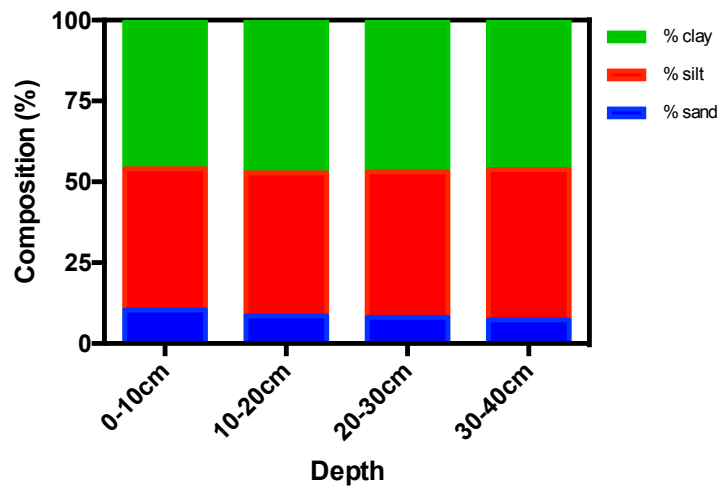

Supplement: Figure S1 — (A) total organic carbon (TOC), (B) soil moisture, (C) soil pH, and (D) particle size. Measurements of TOC, moisture and pH were performed at each depth before and after rain. One inch of rain fell on Day 30 and 3 inches of rain fell on Day 39. TOC decreased by depth and appeared to slightly increase at all depths after the second rainfall. Soil moisture in the upper soil zones was very low before the rainfall and increased with each rain event. Soil pH was fairly uniform between the depths and rose from about 4.6 to 4.9 after the first rain then held steady. The soil was a mixture of clay- and silt-sized particles ( 45% each) with a minor component of sand-sized particles (8–10%). [file peerj-04-2687-s001.pdf]

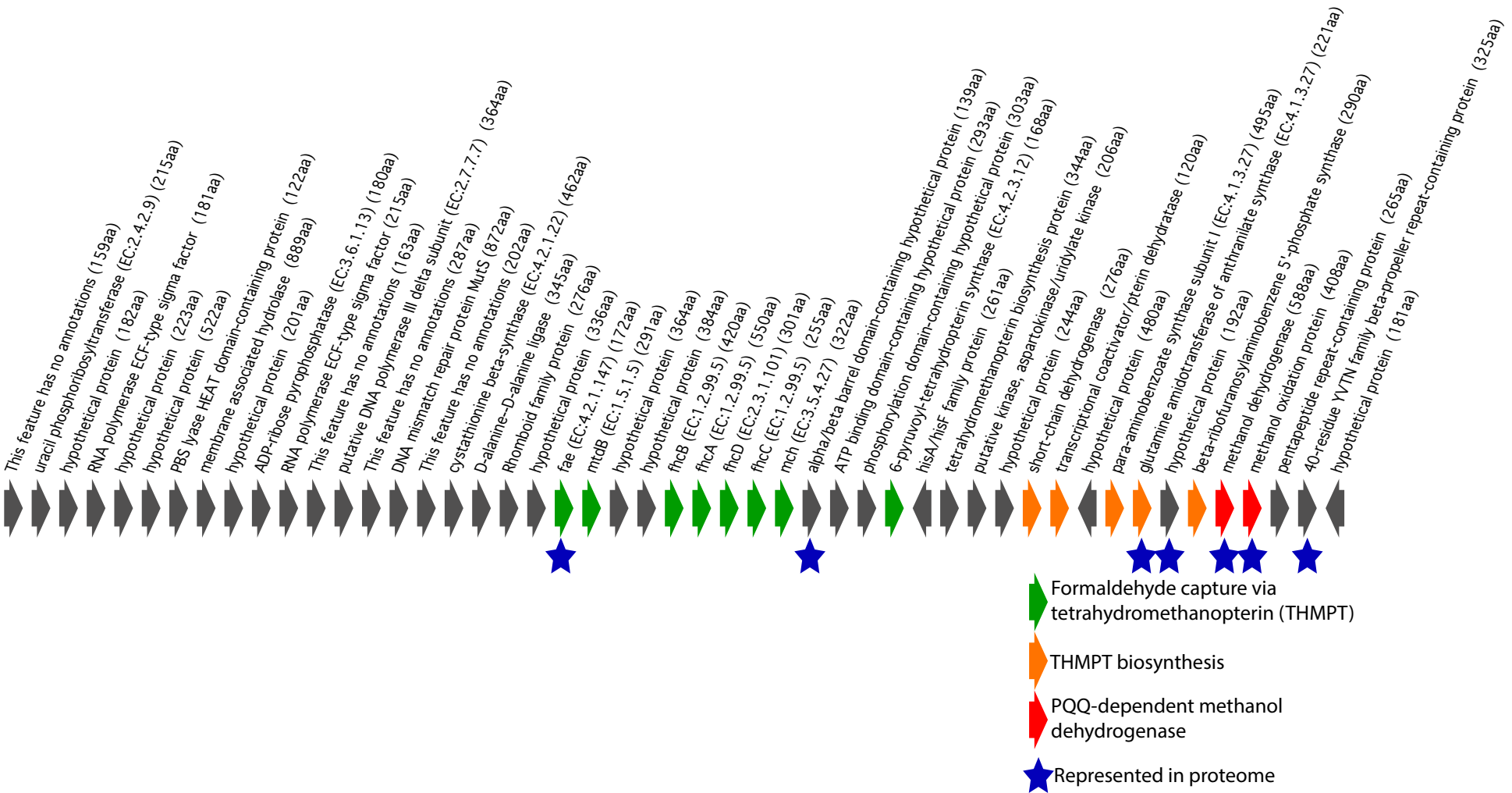

Supplement: Figure S3 — The genetic neighborhood of the methanol dehydrogenase in Gemmatimonadetes encodes for most of the proteins involved in the metabolism of methanol, including the tetrahydromethanopterin (THMPT) biosynthesis (orange), formaldehyde capture (green) pathways, and the methanol dehydrogenase (xoxF) itself and a maturation protein (red). Each of these pathways is represented by at least one known protein in the proteome (indicated by blue star). Gene name abbreviations: formaldehyde activating enzyme (fae); NAD(P)-dependent methylene-tetrahydromethanopterion dehydrogenase (mtdB); formyltransferase/hydroxylating complex (fhc); methenyl-tetrahydromethanopterin cyclohydrolase (mch). [file peerj-04-2687-s003.pdf]

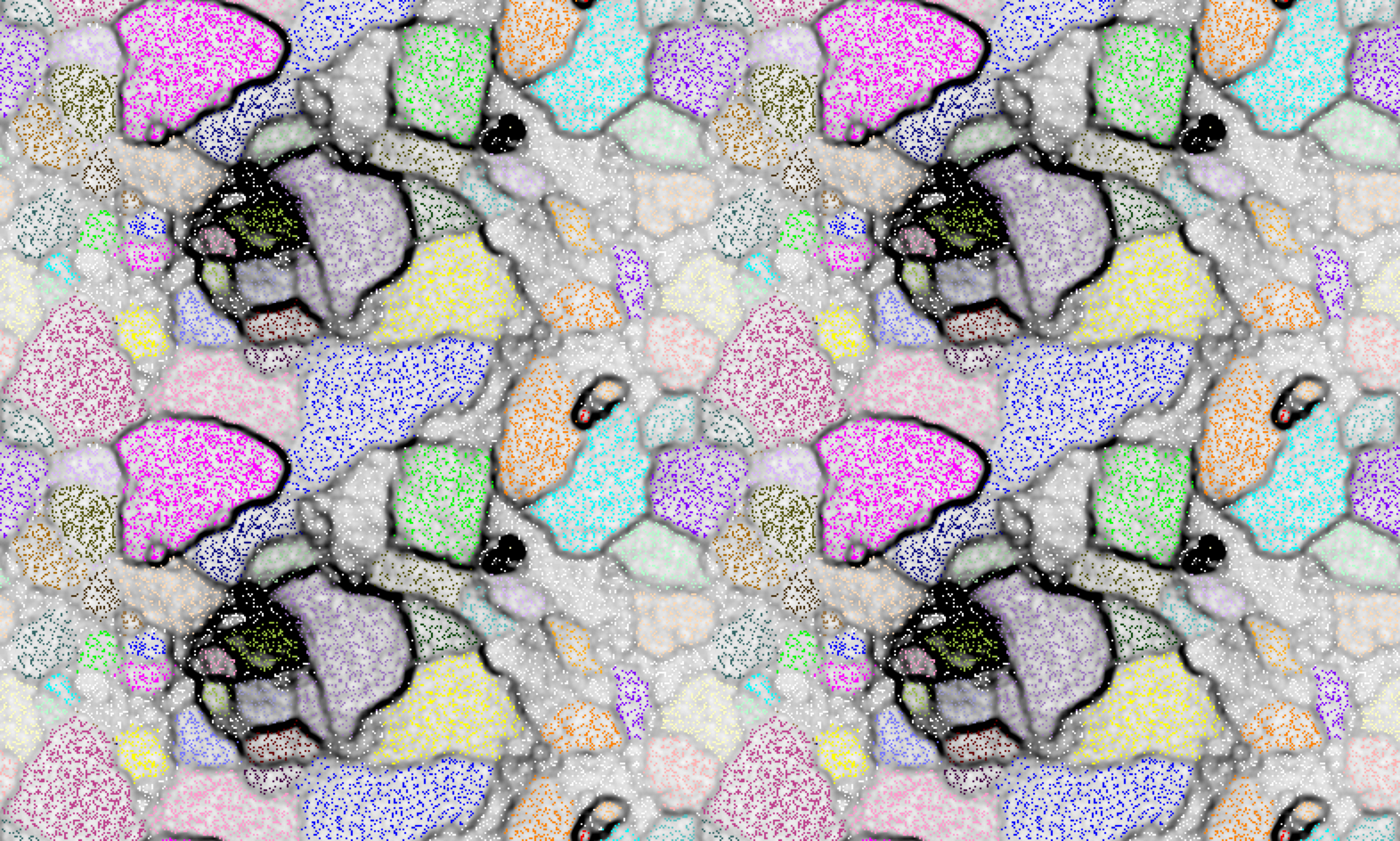

Supplement: Figure S5 — Image of emergent self-organizing map created from time series and coverage patterns of contigs from sample 10–20 cm 2 days after 2nd rain. 48 partial to near-complete genomes were defined (colors were assigned randomly). [file peerj-04-2687-s005.pdf]
