# Supplementary material for: Proteogenomic analyses indicate bacterial methylotrophy and archaeal heterotrophy are prevalent below the grass root zone
Source: PeerJ. 2016 Nov 8;4:e2687. doi: 10.7717/peerj.2687 (PMC5103831; doi:10.7717/peerj.2687)
Supplement: Figure S4 — Rows indicate draft genomes and columns indicate lists of individual genes, with the exception of PQQ biosynthesis where many subunits are represented in a single list. [file peerj-04-2687-s004.pdf]

	PQQ biosynthesis	PQQ-dependent dehydrogenase (MDH)	Formaldehyde activating enzyme (Fae)	Tetrahydromethanopterin N-Formyltransferase (Ftr)	Methenyl-tetrahydromethanopterin cyclohydrolase...	5,10-Methylene-tetrahydromethanopterin Dehydro...	Formate Tetrahydrofolate Ligase (FtfL)	5,10-methylene-tetrahydrofolate dehydrogenase/M...	Methylenetetrahydrofolate dehydrogenase (MtdA)	Serine Cycle-glycine hydroxymethyltransferase (2...	Serine Cycle-alanine-glyoxylate transaminase	Serine Cycle-Glycerate dehydrogenase	Serine Cycle (Gemma) Glycerate Kinase	Serine_Cycle_Phosphoenolpyruvate_carboxylase	Serine Cycle (Gemma)-D-3-phosphoglycerate dehy...	Serine Cycle-malate dehydrogenase 2013	Serine Cycle-malate-CoA ligase	Serine Cycle-malyl-CoA lyase	Serine Cycle-Glycerate 2-kinase	Serine Cycle-enolase	Serine (Roku)_aconitate_hydrolase(4.2.1.3) 2013	Serine (Roku)_isocitrate_lyase(4.1.3.1)	Serine (Roku)_citrate_synthase(2.3.3.1) 2013	Serine (Roku)-isocitrate_dehydrogenase(1.1.1.42)
13_1_20CM_2_Gemmatamonadets-rel_maybe_71-...	1	3	1	2	3	2	1	1	3			1			1	2					1	1		
13_1_20CM_4_Gemmatimonadetes_69_16	3	2	1	1	1	1	1	1	2	1		1	1	2	1	2	2	1		1	1	1	2	2
13_1_20CM_Gemmatimonadetes_69_28	3	1	2	1	1	1	1		1			2		2	1	2	2	1		1		1	1	2
13_1_40CM_2_Gemmatimonadetes_69_13	3	1	1	1	1	1	1	2	3	1	1	3	1	2	1	3	2	1		2	1	1	2	1
13_1_40CM_4_Gemmatimonadetes_69_8	3	4	1	1	1	2	2	2	3	1	1	3	2	2	2	3	2	1		2	1	2	4	2
13_1_40CM_Gemmatimonadetes_69_22_partial	3	2	1	1	1	1	1		1	1	1	1	1	1	1	2	2	1		1	1		2	1
13_2_20CM_2_Gemmatimonadetes_69_23	2	4	1	1	1	1	1	1	1	2	1	3		2	2	4	5	1		3	1		1	3
13_2_20CM_Gemmatimonadetes_69_27	2	2	1	1	1	1	1	1	2	1	1	1		2	1	2	2	1		1	1	2	1	2
13_2_20CM_Gemmatimonadetes_69_8	3	1	1			1		1	2	1		1	1	1	1	2	2	1		1			2	2
13_2_20CM_Gemmatimonadetes_70_9	3	3	1	1	1	1	1		1			1	1	1	1	1	2	1		1	1	2	1	1
13_1_20CM_2_Rokubacteria_68_19	4	1									1	3			3	1				1	1	2	2	
13_1_20CM_4_Rokubacteria_68_9	4	1						1	1	1	2	4		2	3	4	2	2	1	1	3	3	2	1
13_1_20CM_4_Rokubacteria_70_14		2						1	1	2	1	4			3	2			1	1	1	2	3	
13_1_40CM_2_Rokubacteria_68_13	4	1										3		1	2	2					1		2	
13_1_40CM_3_Rokubacteria_69_38		2					1	1	1	1	1	5			3	2			1	1	3	2	4	1
13_1_40CM_4_Rokubacteria_69_39		1					1	1	1	1	1	5			3	3			1	1	4	2	4	1
13_2_20CM_2_Rokubacteria_64_8	4	1						1	1	2		5		2	3	4			1	1	2	3	2	1
13_1_20CM_4_Alphaproteobacteria_65_11											1													
13_1_20CM_2_Alphaproteobacteria_megabin_64_6	20	26				1	8	3		7	4	21		14	19	19	4	12	2	5	13	6	4	9
13_1_20CM_3_Alphaproteobacteria_megabin_62_7	10	5						4			2	4		1	3	4		1	1			1	2	1
13_1_20CM_3_Alphaproteobacteria_megabin_63_6	27	15	1		1		6	11	2	12	8	29	1	13	29	27	5	14	4	10	21	15	8	11
13_1_20CM_4_Alphaproteobacteria_megabin_64_7	27	30					9	5	1	9	8	25		14	34	33	7	10	1	11	18	13	8	15
13_1_20CM_Alphaproteobacteria_megabin_63_14	24	36	1		1		7	10	2	8	4	27		15	25	28	3	9	2	15	17	8	6	19
13_1_40CM_2_Alphaproteobacteria_megabin_64_4	13	26	3	1	1		11	5		5	4	24		17	17	23	13	11	1	8	15	5	7	9
13_1_40CM_3_Alphaproteobacteria_megabin_63_4	12	19		1	1	2	7	4	2	4	4	17		15	21	27	11	13	3	5	10	3	5	4
13_1_40CM_4_Alphaproteobacteria_megabin_63_4	10	21		2		1	7	1		4	1	26		19	11	21	14	11	1	7	8	2	4	6
13_2_20CM_2_Alphaproteobacteria_64_7	2						2	2		2		1			6	4		3	1	1	3	1		1
13_1_20CM_2_Betaproteobacteria_megabin_64_7	13	9		1			8	9	8	7	4	24	1	16	8	16	20	1	3	6	10	7	3	14
13_1_20CM_3_Betaproteobacteria_63_8												1	1	2		1						1		
13_1_20CM_3_Betaproteobacteria_megabin_65_6	5	14					6	7	1	10	2	18		11	5	22	6		2	8	17	17	7	11
13_1_20CM_3_Rhodocyclales_megabin_66_7		2					1		1	1				4	1	2	3			1	1	1		3
13_1_20CM_4_Betaproteobacteria_megabin_65_8	16	12	1		1		7	8	1	9	3	23	3	15	6	20	17	4	2	14	16	14	7	11
13_1_20CM_4_Delftia_acidovorans_67_18		2						1		3	1	11		1	1	4				2	7	1	1	3
13_1_20CM_Betaproteobacteria_67_22																1								
13_1_20CM_Betaproteobacteria_megabin_64_11	7	6					4	5	1	4	5	9	1	3	2	9	6	2		6	5	3	4	8
13_1_20CM_Betaproteobacteria_megabin_65_24		3					3	2		3	1	4		4		4	6	1	1	5	6	4	4	7
13_1_40CM_2_Betaproteobacteria_megabin_64_4	10	8			1		7	9	3	5	5	26		16	9	31	19	7	3	13	16	10	11	13
13_1_40CM_3_Betaproteobacteria_megabin_64_4	15	12	1		1		5	7	7	12	6	21	2	14	4	23	16	1	3	19	19	5	9	15
13_1_40CM_4_Betaproteobacteria_64_4										2		1			1	1				1			1	1
13_1_40CM_4_Betaproteobacteria_megabin_64_5	11	7	1		1	1	8	7	6	8	5	24		19	8	23	11	1		12	21	12	7	12
