# Supplementary material for: Proteogenomic analyses indicate bacterial methylotrophy and archaeal heterotrophy are prevalent below the grass root zone
Source: PeerJ. 2016 Nov 8;4:e2687. doi: 10.7717/peerj.2687 (PMC5103831; doi:10.7717/peerj.2687)
Supplement: Table S2 — (A) Table listing genomes and megabins generated in the study. The summaries of genome characteristics are listed by sample and include genome completeness, size, GC content, and other genome bin quality information. (B) Genomes recovered and the current NCBI and JGI IMG database of genomes for those phyla. [file peerj-04-2687-s007.pdf]

Created in ggKbase on 9/22/2015

Pre-rain plot 1 10 - 20 cm

Organism name	bin length	Partial to near complete	Bins/Megabins	GC%	coverage	# contigs	# features	longest contig	RP Inventory (total: 55)	RP multiple	BSCG Inventory (total: 51)	BSCG multiple	ASCG Inventory (total: 38)	ASCG multiple	completion status
13_1_20cm_UNK	2.72E+08			60.56	14.73	132399	383256	58324	54	53	51	51	38	38	megabin
13_1_20cm_virus_1	4648			42.62	67745.32	1	7	4648	0	0	0	0	0	0	partial
13_1_20CM_Alphaproteobacteria_megabin_63_14	38528272			62.83	13.9	20676	51307	21416	53	52	51	51	13	12	megabin
13_1_20CM_Choroflexi_megabin_53_42	4859784			53.05	42.11	2203	5680	8307	39	13	37	17	13	8	partial
13_1_20CM_Chloroflexi_megabin_67_15	11414150			67.09	15.55	6586	14173	10021	34	22	40	28	12	8	partial
13_1_20CM_Chloroflexi_megabin_53_13	25232114			52.74	13.29	13569	31767	24402	51	50	47	44	15	14	megabin
13_1_20CM_Gemmatimonadetes_megabin_68_21	14062384			67.75	22.04	6294	17231	49383	53	52	50	49	15	13	megabin
13_1_20CM_Verrucomicrobia_megabin_56_12	17936976			55.91	12.87	9427	23553	18202	50	50	50	49	15	14	megabin
13_1_20CM_Acidobacteria_megabin_56_12	27798107			55.91	12.77	14189	33235	25502	53	53	51	51	16	16	megabin
13_1_20CM_Acidobacteria_megabin_65_14	7795168			64.66	13.55	4266	9223	15279	51	48	49	44	14	7	megabin
13_1_20CM_Methylomirabilis_megabin_70_15	15032576			69.77	14.86	6709	20690	18460	52	49	51	47	15	14	megabin
13_1_20CM_Actinobacteria_megabin_69_28	9458937			68.9	27.49	4939	12358	13300	52	52	51	50	16	14	megabin
13_1_20CM_Actinobacteria_megabin_70_12	71381526			69.65	12.41	37007	98427	28278	52	52	51	51	18	17	megabin
13_1_20CM_Deltaproteobacteria_megabin_68_15	17161323			68.06	14.96	9128	21728	17333	51	51	49	48	14	12	megabin
13_1_20CM_Betaproteobacteria_megabin_65_24	6515059			65.28	23.55	2870	8346	18745	51	46	51	48	15	12	megabin
13_1_20CM_Betaproteobacteria_megabin_64_11	13286508			63.93	11.15	7171	17909	20689	50	45	50	44	16	12	megabin
13_1_20CM_Gemmatimonadetes_69_28	2375461	2375461		68.84	28.09	154	2332	41155	9	1	9	1	4	1	partial
13_1_20CM_Chloroflexi_66_33	793227	793227		66.32	33.26	64	815	37216	24	0	12	0	2	0	partial
13_1_20CM_Archaea_52_20	762164	762164		52.18	20.25	69	801	28724	17	2	4	0	16	0	partial
13_1_20CM_Methylomirabilis_70_15	912272	912272		69.59	15.42	84	1004	22951	4	0	2	0	0	0	partial
13_1_20CM_Chloroflexi_54_36	1338217	1338217		54.37	36.24	117	1349	30171	24	0	22	0	2	0	partial
13_1_20CM_Chloroflexi_50_12	540597	540597		49.94	11.85	49	616	51310	21	0	16	0	1	0	partial
13_1_20CM_Betaproteobacteria_67_22	169873	169873		67.09	22.32	18	194	13809	18	1	19	0	1	0	partial
13_1_20CM_Acidobacteria_58_21	3920195	3920195		57.88	20.66	240	3510	51722	33	0	31	1	9	1	partial
13_1_20CM_Verrucomicrobia_54_28	2281644	2281644		54.14	28.07	119	2181	48122	42	1	37	0	8	0	partial
13_1_20CM_Gemmatimonadetes_66_74_partial	53692			66.33	75.08	25	62	9386	16	1	15	0	2	0	partial
13_1_20CM_Gemmatimonadetes_69_60_partial	120514			68.75	59.33	70	161	10017	18	0	18	0	2	0	partial
13_1_20CM_Gemmatimonadetes_69_52_partial	202485			69	52.54	95	273	13883	18	0	19	2	1	1	partial
Sum	5.66E+08	13093650	2%												
Count	28	9	32%												

Four days after first rain plot 1 10 - 20 cm

Organism name	bin length	Partial to near complete	Bins/Megabins	GC%	coverage	# contigs	# features	longest contig	RP Inventory (total: 55)	RP multiple	BSCG Inventory (total: 51)	BSCG multiple	ASCG Inventory (total: 38)	ASCG multiple	completion status
13_1_20CM_2_UNK	3.02E+08			62.48	9.24	129447	419029	101778	54	53	51	51	38	35	megabin
13_1_20CM_2_Archaea_Thermoplasmatales_64_100	253661			64.17	91.68	95	349	16827	22	1	6	0	21	1	partial
13_1_20CM_2_Thaumarchaeota_39_11	451093			39.35	18.63	134	606	7908	3	0	1	0	15	3	partial
13_1_20CM_2_Thaumarchaeota_38_5	442543			37.95	8.59	144	577	7895	2	0	0	0	11	3	partial
13_1_20CM_2_Gemmatamonadets-rel_maybe_71-24_part	964988			70.54	41.03	307	1320	15349	8	1	7	1	3	0	partial
13_1_20CM_2_Novel_55_9	1736449			54.67	15.67	452	2153	16489	13	2	4	0	2	0	partial
13_1_20CM_2_Deltaproteobacteria_megabin_68_13	1862494			68.13	21.42	737	2125	14005	20	2	19	2	7	3	partial
13_1_20CM_2_Archaea_51_12	3289518			51.61	20.05	483	3973	61193	10	0	5	2	12	3	partial
13_1_20CM_2_Archaea_MCG_51_8	3613112			51.58	13.71	944	4711	71911	9	2	4	1	11	4	partial
13_1_20CM_2_Deltaproteobacteria_megabin_68_7	19570670			67.95	7.46	11702	24471	17114	49	44	48	43	13	12	megabin
13_1_20CM_2_Deltaproteobacteria_megabin_56_5	7146664			55.8	5.44	4267	8477	20081	42	33	44	35	11	7	megabin
13_1_20CM_2_Alphaproteobacteria_megabin_64_6	25684884			64.28	6.97	15877	34007	18020	47	39	45	37	13	9	megabin
13_1_20CM_2_Betaproteobacteria_megabin_64_7	22582789			64.26	7.33	13565	30524	17643	52	51	51	51	15	14	megabin
13_1_20CM_2_Gammaproteobacteria_megabin_63_6	13185561			62.88	6.65	8426	16126	11871	15	5	15	9	7	3	partial
13_1_20CM_2_Proteobacteria_megabin_65_9	21830755			64.88	9.44	7672	30925	41238	42	21	34	19	11	6	partial
13_1_20CM_2_Gemmatimonadetes_megabin_67_6	8316215			67.1	7.03	4159	10294	36460	52	48	49	47	13	12	megabin
13_1_20CM_2_Actinobacteria_megabin_66_6	32885460			66.32	6.41	20276	44293	19608	51	51	51	51	17	14	megabin
13_1_20CM_2_Gemmatimonadetes_megabin_69_23	8618661			68.97	20.82	2879	10148	47444	53	51	51	50	13	12	megabin
13_1_20CM_2_Acidobacteria_megabin_59_6	39710864			58.63	6.83	21029	45978	31137	53	52	51	51	15	15	megabin
13_1_20CM_2_Firmicutes_megabin_67_9	13692972			67.34	9	8201	18110	21641	52	52	50	50	19	14	megabin
13_1_20CM_2_Chloroflexi_megabin_67_8	13116783			66.97	7.99	7607	16268	36828	40	18	39	19	12	11	partial
13_1_20CM_2_Nitrospirae_megabin_62_12	1607493			63.25	32.08	835	2241	8463	39	21	39	17	7	3	partial
13_1_20CM_2_Verrucomicrobia_megabin_58_5	15474218			58.18	5.1	8958	19382	12700	51	50	49	49	15	15	megabin
13_1_20CM_2_Firmicutes_megabin_55_6	4924225			55.07	6.28	3140	6001	7828	33	11	32	14	9	6	partial
13_1_20CM_2_Chloroflexi_megabin_53_5	7640243			53.21	4.65	4855	9943	14029	40	22	37	24	13	7	partial
13_1_20CM_2_Archaea_megabin_64_15	14674057			63.83	15.73	7458	19308	22411	41	38	29	25	38	38	megabin
13_1_20CM_2_Archaea_megabin_51_10	20834786			50.75	10.15	7736	25500	107600	41	38	29	25	38	38	megabin
13_1_20CM_2_Nitrospirae_megabin_63_13	1373296			62.17	12.99	553	1666	27881	30	4	32	5	4	0	partial
13_1_20CM_2_Nitrospirae_megabin_61_5	3714973			60.51	5.11	2277	4789	8299	31	3	35	8	9	5	partial
13_1_20CM_2_Novel_70_7	2175604	2175604		69.35	6.75	165	2380	40659	17	1	11	0	7	1	partial
13_1_20CM_2_Rokubacteria_69_58	2617030	2617030		69.97	58.53	375	2987	41392	46	1	46	3	11	1	near
13_1_20CM_2_Novel_70_9	1564845	1564845		70.3	8.54	109	1677	59505	28	0	31	0	10	0	partial
13_1_20CM_2_Novel_68_14	2223188	2223188		67.98	13.83	141	2098	82545	15	0	15	1	7	0	partial
13_1_20CM_2_Deltaproteobacteria_69_21	2852113	2852113		68.46	21.21	187	2899	41229	30	1	29	3	6	0	partial
13_1_20CM_2_Actinobacteria_66_18	1426442	1426442		65.94	18.13	104	1516	42156	19	0	19	0	4	0	partial
13_1_20CM_2_Novel_65_9	4585906	4585906		64.87	9.32	313	4177	41922	13	1	12	0	7	0	partial
13_1_20CM_2_Actinobacteria_65_11	2460926	2460926		65.31	11	96	2608	97298	17	1	18	0	9	0	partial
13_1_20CM_2_Acidobacteria_60_10	2172905	2172905		60.02	10.09	166	2006	32164	32	0	36	0	10	1	partial
13_1_20CM_2_Acidobacteria_55_15	3487967	3487967		55.45	15.48	211	3457	61806	45	0	46	0	14	0	near
13_1_20CM_2_Novel_68_7	1665476	1665476		67.93	6.77	137	1544	30448	12	1	11	0	4	1	partial
13_1_20CM_2_Gemmatimonadetes_70_10	1584817	1584817		70.04	10.12	98	1619	49180	20	0	22	3	8	0	partial
13_1_20CM_2_Nitrospirae_62_14	1028956	1028956		62.21	13.96	74	1053	41113	11	0	10	0	8	0	partial
13_1_20CM_2_Acidobacteria_57_8	1112052	1112052		56.53	7.59	105	1135	21774	21	0	20	0	5	0	partial
13_1_20CM_2_Chloroflexi_59_7	1808416	1808416		58.68	6.63	154	1800	27929	34	3	31	2	7	1	partial
13_1_20CM_2_Rokubacteria_68_19	2818324	2818324		68.3	19.24	186	3054	56673	22	1	26	1	11	0	partial
13_1_20CM_2_Archaea_megabin_38_9	1136162	1136162		37.8	9.26	68	1347	52505	30	2	12	0	25	1	partial
13_1_20CM_2_Archaea_SAGMCG_39_20	1067158	1067158		39.02	19.75	61	1255	62635	30	1	9	1	18	0	partial
13_1_20CM_2_Archaea_MCG_53_14	1549201	1549201		53.3	14.09	42	1657	103740	31	4	12	0	23	2	partial
13_1_20CM_2_Archaea_54_9.0_partial	1900675			53.38	9.67	116	2036	116279	15	1	5	1	15	0	partial
Sum	6.53E+08	39337488	6%												
Count	49	19	39%												

Six days after first rain plot 1 10 - 20 cm

Organism name	bin length	Partial to near complete	Bins/Megabins	GC%	coverage	# contigs	# features	longest contig	RP Inventory (total: 55)	RP multiple	BSCG Inventory (total: 51)	BSCG multiple	ASCG Inventory (total: 38)	ASCG multiple	completion status
13_1_20cm_3_UNK	2.4E+08			61.31	7.11	101274	335013	118830	55	53	51	51	25	19	megabin
13_1_20CM_3_Gemmatimonas_aurantiaca_60_15	897127			60.28	14.51	298	1066	7995	35	1	30	0	8	2	partial
13_1_20CM_3_Alphaproteobacteria_megabin_62_7	5391795			61.82	7.6	2026	7983	9288	41	30	33	13	10	7	partial
13_1_20CM_3_Rhodocyclales_megabin_66_7	2155558			65.94	7.37	1199	2932	12318	40	16	35	14	8	3	partial
13_1_20CM_3_Actinobacteridae_megabin_60_6	68596191			69.03	6.76	35763	92249	25710	52	52	51	51	18	17	megabin
13_1_20CM_3_Alphaproteobacteria_megabin_63_6	38116484			63.32	6.71	22501	50498	15190	53	53	51	51	15	13	megabin
13_1_20CM_3_Actinobacteria_megabin_70_7	45061265			70.04	7.59	21646	63851	53340	52	52	51	51	18	17	megabin
13_1_20CM_3_Betaproteobacteria_megabin_65_6	20205575			64.94	6.92	10750	27317	21724	52	48	50	44	16	14	megabin
13_1_20CM_3_Deltaproteobacteria_megabin_65_6	21447536			65.15	6.51	11976	26344	22151	53	49	51	46	14	11	megabin
13_1_20CM_3_Fungi_48_4	18107678			47.69	4.47	8966	20345	23198	30	10	5	2	17	10	partial
13_1_20CM_3_Chloroflexi_megabin_53_7	15700182			52.95	7.51	8195	19393	8177	52	48	45	41	14	12	megabin
13_1_20CM_3_Verrucomicrobia_megabin_56_15	2068733			56.01	14.56	767	2460	8730	44	26	35	17	13	5	partial
13_1_20CM_3_Acidobacteria_megabin_58_5	35100898			58.25	5.43	19828	43199	11581	53	53	51	49	17	15	megabin
13_1_20CM_3_Verrucomicrobia_megabin_57_6	32748512			56.74	5.95	16770	42189	21688	51	51	50	49	17	17	megabin
13_1_20CM_3_Proteobacteria_megabin_63_7	24206792			63.57	7.49	11427	32626	39168	36	29	33	23	12	10	partial
13_1_20CM_3_Gemmatimonadetes_megabin_65_8	17202658			64.7	7.94	7254	21168	38437	53	53	51	51	16	15	megabin
13_1_20CM_3_Chloroflexi_megabin_68_7	17201551			67.52	7.06	9527	21527	16209	49	45	48	46	14	10	megabin
13_1_20CM_3_Firmicutes_megabin_68_7	8490665			67.41	7.33	5095	11160	22014	50	48	50	47	14	10	megabin
13_1_20CM_3_Methylomirabilis_megabin_69_6	8575535			69.06	6.55	4432	11774	13509	46	43	43	37	12	9	megabin
13_1_20CM_3_Cyanobacteria_megabin_60_6	10051784			60.02	5.88	6146	11385	34186	4	2	4	0	4	1	partial
13_1_20CM_3_Bacteriodetes_megabin_54_5	9600510			54.08	5.43	5714	11243	10736	39	13	34	8	11	5	partial
13_1_20CM_3_Archaea_megabin_66_7	2790111			65.21	7.54	1594	3711	12970	32	24	15	8	34	20	megabin
13_1_20CM_3_Archaea_megabin_53_6	1513093			52.45	5.93	761	1897	13374	30	11	9	1	35	9	megabin
13_1_20CM_3_Archaea_megabin_36_3	1143233			36.23	3.26	779	1590	4664	16	3	3	1	17	7	partial
13_1_20CM_3_Actinobacteria_68_10	1191042	1191042		67.88	10.36	98	1325	26599	7	0	7	0	3	0	partial
13_1_20CM_3_Actinobacteria_71_11	6222268	6222268		71.18	11.36	444	6432	49005	42	4	35	4	12	5	partial
13_1_20CM_3_Chloroflexi_54_15	3194828			53.56	14.5	259	3112	30397	32	0	29	2	8	2	partial
13_1_20CM_3_Acidobacteria_58_11	2802244	2802244		58.54	11.42	199	2620	43135	16	2	12	0	5	0	partial
CONTAMINANT_13_1_20CM_3_Alphaproteobacteria_64_12	1874975	1874975		64.11	11.6	162	1975	27149	33	1	28	0	5	0	partial
13_1_20CM_3_Actinobacteria_68_9	2365548	2365548		68.89	8.68	152	2555	65518	46	1	46	1	9	1	near
13_1_20CM_3_Actinobacteria_70_7	5807638	5807638		69.84	6.89	415	5747	48528	36	5	31	3	8	2	partial
13_1_20CM_3_Betaproteobacteria_63_8	1956095	1956095		62.6	7.85	167	2069	27067	36	3	34	3	7	2	partial
13_1_20CM_3_Acidobacteria_53_8	6121216	6121216		52.84	8.55	155	5562	252073	48	1	48	0	14	1	near
13_1_20CM_3_Verrucomicrobia_54_17	2455393	2455393		54.18	16.89	143	2436	67045	40	1	35	0	10	0	partial
Sum	6.8E+08	30796419	5%												
Count	34	9	26%												

2 days after second rain plot 1 10 - 20 cm

Organism name	bin length	Partial to near complete	Bins/Megabins	GC%	coverage	# contigs	# features	longest contig	RP Inventory (total: 55)	RP multiple	BSCG Inventory (total: 51)	BSCG multiple	ASCG Inventory (total: 38)	ASCG multiple	completion status
13_1_20cm_4_UNK	1.85E+08			63	7.77	80947	265900	97665	53	53	51	51	30	22	megabin
13_1_20CM_4_Delftia_acidovorans_67_18	6412626			66.65	29.66	53	5784	538623	52	1	51	0	13	0	near
13_1_20CM_4_Ktedonobacteria_53_7	1539877			52.68	11.32	448	1651	8036	10	0	9	0	4	0	partial
13_1_20CM_4_Rokubacteria_70_13	650906			70.38	13.21	256	814	12613	22	3	25	3	4	2	partial
13_1_20CM_4_GAL15_megabin_65_7	2394148			65.04	6.52	837	2904	17102	47	12	44	15	10	8	megabin
13_1_20CM_4_Nitrospirae_megabin_61_6	6128098			60.64	6.51	2509	7580	19329	46	38	47	38	14	11	megabin
13_1_20CM_4_Alphaproteobacteria_megabin_64_7	36316066			63.59	7.19	21492	48762	18319	53	52	51	49	11	11	megabin
13_1_20CM_4_Actinobacteria_megabin_69_7	59267558			69.03	6.86	33556	81766	19964	53	51	51	51	17	17	megabin
13_1_20CM_4_Euryarchaeota_megabin_65_10	4239442			64.58	10.95	2173	5487	36829	37	30	21	14	38	32	megabin
13_1_20CM_4_Euryarchaeota_52_7	1565737			52.36	7.57	883	1897	18873	22	12	7	3	21	16	partial
13_1_20CM_4_Archaea_megabin_64_10	1174542			63.57	10.5	535	1488	43329	4	0	4	1	13	4	partial
13_1_20CM_4_Archaea_megabin_52_8	3748098			51.85	8.1	1490	4742	24296	36	31	16	11	36	32	megabin
13_1_20CM_4_Archaea_megabin_37_4	1442506			36.21	4.82	603	2057	24383	31	5	12	6	37	12	megabin
13_1_20CM_4_Deltaproteobacteria_megabin_68_7	19760215			68.48	8	9844	24206	36640	52	49	50	47	17	13	megabin
13_1_20CM_4_Betaproteobacteria_megabin_65_8	21891023			64.82	8.29	12916	29846	23303	52	51	51	50	15	13	megabin
13_1_20CM_4_Acidobacteria_megabin_58_6	45609096			57.85	6.17	25146	55572	17558	53	53	51	51	16	15	megabin
13_1_20CM_4_Proteobacteria_megabin_63_8	29093346			63.03	7.86	14972	38684	41144	44	32	43	37	17	14	megabin
13_1_20CM_4_Gemmatimonadetes_megabin_67_9	17470607			66.68	9.15	8084	21615	37703	53	53	51	50	16	14	megabin
13_1_20CM_4_Methylomirabilis_megabin_69_7	17403923			69.5	7.65	7910	23267	33658	51	50	50	48	14	13	megabin
13_1_20CM_4_Verrucomicrobia_megabin_57_6	20520797			56.67	6.27	11859	26742	14913	51	50	50	49	16	15	megabin
13_1_20CM_4_Chloroflexi_megabin_62_6	21422472			62.39	6.61	12661	27092	12562	49	48	50	49	14	12	megabin
13_1_20CM_4_Firmicutes_megabin_64_7	9992075			64.26	7.32	6345	13076	13274	50	44	49	46	16	12	megabin
13_1_20CM_4_Bacteriodetes_60_6	5944011			60.62	6.87	3728	6853	25239	12	1	11	2	5	1	partial
13_1_20CM_4_Cyanobacteria_61_6	5758731			60.73	6.14	3730	6540	9277	6	2	5	1	2	1	partial
13_1_20CM_4_Rokubacteria_megabin_66_7	4198491			66.63	7.2	2477	5355	10572	13	7	16	9	8	3	partial
13_1_20CM_4_Fungi_44_6	3006783			41.91	6.32	1892	3569	18980	5	1	1	0	4	2	partial
13_1_20CM_4_virus_53_5	17017			51.89	4.17	12	20	2309	0	0	0	0	0	0	partial
13_1_20CM_4_Gemmatimonadetes_69_16	2835016	2835016		69.13	15.82	88	2736	162773	30	1	26	0	10	0	partial
13_1_20CM_4_Rokubacteria_68_9	4240272	4240272		68.29	9.48	240	4636	124098	50	2	45	1	12	0	near
13_1_20CM_4_Verrucomicrobia_54_11	2851651	2851651		54.28	11.19	168	2830	65602	45	4	44	3	12	3	near
13_1_20CM_4_Chloroflexi_53_11	2649496	2649496		53.43	10.83	212	2525	35378	39	1	36	1	10	3	partial
13_1_20CM_4_Acidobacteria_57_11	2007395	2007395		56.79	10.59	164	1908	50789	1	0	3	1	2	1	partial
13_1_20CM_4_Chloroflexi_66_7	652077	652077		66.27	6.79	64	700	19342	15	0	18	0	3	0	partial
13_1_20CM_4_Gemmatimonadetes_66_11	3107513	3107513		65.85	10.97	120	3112	132319	43	0	43	2	10	0	near
13_1_20CM_4_Archaea_Thermoplasmatales_42_14	998369	998369		63.74	14.37	76	1124	43307	24	2	7	0	23	1	partial
13_1_20CM_4_Rokubacteria_70_14	3352455	3352455		69.93	14.22	177	3560	73047	36	3	36	2	10	0	partial
13_1_20CM_4_Actinobacteria_68_12	1425745	1425745		68.3	12.33	109	1545	29921	9	0	11	0	6	0	partial
13_1_20CM_4_Acidobacteria_56_7	2987195	2987195		55.52	6.97	239	2968	43113	44	10	40	6	12	4	partial
13_1_20CM_4_Verrucomicrobia_55_9	590700	590700		55.37	9.22	53	617	16557	15	2	9	1	4	0	partial
13_1_20CM_4_Actinobacteria_69_9	1895327	1895327		69.48	9.42	135	2236	37235	31	1	35	1	12	1	partial
13_1_20CM_4_Alphaproteobacteria_65_11	584984	584984		65.46	10.81	48	651	24206	28	0	26	0	3	0	partial
13_1_20CM_4_Armatimonadetes_65_7	197337	197337		64.5	6.74	18	216	16301	19	0	19	0	1	0	partial
13_1_20CM_4_Novel_66_15	251877	251877		65.96	15.27	24	300	19140	21	0	18	0	0	0	partial
13_1_20CM_4_Nitrospirae_62_6	285956	285956		62.32	5.76	26	324	16465	18	0	18	0	1	0	partial
13_1_20CM_4_Chloroflexi_66_15	387149	387149		65.65	15.19	35	433	20642	15	0	16	0	3	0	partial
Sum	5.63E+08	31300514	6%												
Count	45	18	40%												

Six days after first rain plot 2 10 - 20 cm

Organism name	bin length	Partial to near complete	Bins/Megabins	GC%	coverage	# contigs	# features	longest contig	RP Inventory (total: 55)	RP multiple	BSCG Inventory (total: 51)	BSCG multiple	ASCG Inventory (total: 38)	ASCG multiple	completion status
13_2_20cm_UNK	3.94E+08			61.94	7.45	182445	519322	136805	53	53	51	51	38	38	megabin
13_2_20CM_Acidobacteria_56_17	1281942			56.43	23.32	388	1494	41408	13	0	16	0	4	0	partial
13_2_20CM_Ktedonobacter_racemifer_53_11	1381286			53.43	11.12	424	1459	7981	4	0	6	0	3	0	partial
13_2_20CM_Actinobacteria_69_6	53712720			69.07	6.7	28950	72879	25639	52	52	51	51	17	16	megabin
13_2_20CM_Nitrospirae_61_6	3544200			61.73	7.08	1489	4388	45398	48	34	48	35	14	7	megabin
13_2_20CM_Chloroflexi_53_11	4013008	4013008		53.4	11.09	291	3805	52554	46	1	42	3	12	1	near
13_2_20CM_Gemmatimonadetes_69_27	2728361			69.04	26.77	132	2625	81369	22	3	21	0	7	0	partial
13_2_20CM_Gemmatimonadetes_70_9	2336631	2336631		69.73	9.08	138	2313	55958	21	2	17	2	7	0	partial
13_2_20CM_Verrucomicrobia_54_12	2889335	2889335		54.46	12.3	173	2821	106059	49	1	45	2	11	0	near
13_2_20CM_Rokubacteria_70_12	2180659	2180659		69.87	11.9	166	2408	37039	30	1	27	0	7	0	partial
13_2_20CM_Gammaproteobacteria_66_19	3146190	3146190		68.14	19.59	136	2975	88571	41	2	42	2	12	1	near
13_2_20CM_Acidobacteria_57_17	4590159	4590159		57	16.82	191	4182	133163	34	1	33	0	10	0	partial
13_2_20CM_Gemmatimonadetes_69_8	1996042	1996042		69.07	7.84	130	2024	45131	19	1	16	0	9	0	partial
13_2_20CM_Acidobacteria_58_27	7421935	7421935		58.03	26.71	205	6504	210655	50	0	47	0	14	1	near
13_2_20CM_Rokubacteria_69_15	3214421	3214421		69.46	14.66	151	3323	80498	48	2	46	1	12	0	near
13_2_20CM_Acidobacteria_55_8	2842013	2842013		55.3	7.93	168	2789	56763	32	0	28	0	10	0	partial
13_2_20CM_Acidobacteria_57_7	2945539	2945539		57.37	6.91	189	2835	56287	28	0	26	2	7	0	partial
13_2_20CM_Actinobacteria_68_14	1648675	1648675		68.07	13.62	117	1822	36974	42	1	43	1	10	0	near
13_2_20CM_Rokubacteria_69_10	2286599	2286599		68.78	9.51	111	2471	68143	34	1	28	0	6	0	partial
13_2_20CM_Verrucomicrobia_55_10	2200413	2200413		54.52	9.58	139	2268	53677	44	3	40	1	11	0	partial
13_2_20CM_Rokubacteria19cls_69_15	1844157	1844157		69.35	14.49	123	1999	49484	37	1	35	0	8	0	partial
13_2_20CM_Nitrospira_62_7	1463348	1463348		61.87	6.62	107	1499	33478	14	0	15	1	7	0	partial
13_2_20CM_Gemmatimonadetes__33_partial	487410			69.55	32.5	172	565	14706	19	0	19	0	4	1	partial
13_2_20CM_Gemmatimonadetes__27_partial	372139			68.85	26.76	129	419	9721	15	0	17	0	5	1	partial
Sum	5.04E+08	47019124	9%												
Count	24	16	67%												

2 days after second rain plot 2 10 - 20 cm

Organims name	bin length	Partial to near complete	Bins/Megabins	GC%	coverage	# contigs	# features	longest contig	RP Inventory (total: 55)	RP multiple	BSCG Inventory (total: 51)	BSCG multiple	ASCG Inventory (total: 38)	ASCG multiple	completion status
13_2_20cm_2_UNK	5.41E+08			62.18	5.15	248891	716521	101745	54	54	51	51	38	38	megabin
13_2_20CM_2_Actinobacteria_69_4	72013429			69.04	4.69	37343	95972	62886	52	52	51	51	16	16	megabin
13_2_20CM_2_Ktedonobacter_racemifer_54_8	2225034			53.74	8.42	693	2428	8316	36	2	29	1	6	0	partial
13_2_20CM_2_Nitrospirae_63_8	1199149			63.28	7.61	327	1454	21224	25	1	18	1	8	0	partial
13_2_20CM_2_Nitrospirae_61_4	3251986			60.57	3.97	1899	4284	8951	40	12	33	8	9	2	partial
13_2_20CM_2_Chloroflexi_68_5	16023957			67.56	4.85	8999	19794	20083	50	43	49	44	14	11	megabin
13_2_20CM_2_Chloroflexi_52_3	11782418			51.79	3.53	7196	14921	11142	49	37	45	28	13	9	megabin
13_2_20CM_2_Rokubacteria_70_11	2941729	2941729		69.77	10.7	157	3087	73031	41	2	42	0	11	0	near
13_2_20CM_2_Verrucomicrobia_54_15	1515335	1515335		54.06	15.28	101	1519	41435	23	1	16	0	6	0	partial
13_2_20CM_2_Chloroflexi_56_8	2919941	2919941		53.51	8.13	224	2802	53893	13	0	12	1	9	1	partial
13_2_20CM_2_Acidobacteria_57_12	2402506	2402506		56.75	11.72	176	2299	61786	10	0	10	0	6	0	partial
13_2_20CM_2_Nitrospirae_62_8	2091469	2091469		61.74	7.77	86	2077	74844	44	0	41	0	10	0	near
13_2_20CM_2_Rokubacteria_64_8	3686488	3686488		68.42	8.33	201	3993	84354	43	3	43	0	13	0	near
13_2_20CM_2_Verrucomicrobia_9cls_54_15	952975	952975		54.35	15.01	73	984	40728	26	0	24	0	5	2	partial
13_2_20CM_2_Archaea_52_21	2328907	2328907		51.89	20.78	216	2534	125287	27	13	8	2	33	10	megabin
13_2_20CM_2_Archaea_53_6	1797420	1797420		53.25	6.19	69	1937	107814	31	5	11	0	24	1	partial
13_2_20CM_2_Gemmatimonadetes_65_7	2720911	2720911		65.06	6.77	101	2703	95892	41	1	38	0	8	0	partial
13_2_20CM_2_Gemmatimonadetes_66_5	1368108	1368108		65.93	4.56	99	1409	46541	23	0	20	0	6	0	partial
13_2_20CM_2_Actinobacteria_72_6	2976603	2976603		72.08	6.2	214	3048	54296	7	0	7	0	7	2	partial
13_2_20CM_2_Acidobacteria_66_4	2932631	2932631		65.75	4.17	223	2801	35293	15	0	14	0	5	0	partial
13_2_20CM_2_Acidobacteria_57_6	4478103	4478103		57.37	6.46	169	4083	101462	32	0	29	0	11	2	partial
13_2_20CM_2_Gemmatimonadetes_69_23	3898014	3898014		69.02	22.57	787	4270	77916	46	9	41	2	12	1	near
13_2_20CM_2_Actinobacteria_71_6	5848902	5848902		70.86	5.69	282	5689	93156	9	1	13	0	10	2	partial
13_2_20CM_2_Actinobacteria_66_6	8375137	8375137		66.48	6.28	578	9134	93247	48	38	48	37	12	10	megabin
13_2_20CM_2_Alphaproteobacteria_64_7	6556564	6556564		63.74	7.4	359	6721	116881	49	30	50	30	11	5	megabin
13_2_20CM_2_Acidobacteria_55_5	1813282	1813282		55.19	5	133	1861	32052	13	0	7	0	9	2	partial
Sum	7.1E+08	61605025	9%												
Count	26	19	73%												

Pre-rain plot 1 30 - 40 cm

Organism name	bin length	Partial to near complete	Bins/Megabins	GC%	coverage	# contigs	# features	longest contig	RP Inventory (total: 55)	RP multiple	BSCG Inventory (total: 51)	BSCG multiple	ASCG Inventory (total: 38)	ASCG multiple	completion status
13_1_40cm_UNK	5.61E+08			61.99	11.57	236739	732455	98317	54	54	51	51	38	38	megabin
13_1_40CM_Deltaproteobacteria_57_7	11582400			56.68	6.84	6398	14500	18082	50	40	48	38	14	10	megabin
13_1_40CM_Arch_SAGMCG_38_12	1523451	1523451		37.8	12.08	93	1713	51905	30	3	11	1	32	2	near
13_1_40CM_Rokubacteria_69_96	1680494	1680494		69.25	96.1	380	2023	19210	42	1	37	0	10	0	partial
13_1_40CM_Chloroflexi_66_19	1856841	1856841		65.62	18.53	117	2011	53733	40	1	42	0	9	1	near
13_1_40CM_Acidobacteria_56_16	4636425	4636425		55.74	15.68	261	4566	68133	47	2	47	1	14	1	near
13_1_40CM_Rokubacteria_69_27	3672185	3672185		69.26	27.69	127	3813	142874	49	4	46	0	14	0	near
13_1_40CM_Rokubacteria_68_15	4138625	4138625		68.32	15.22	174	4420	87893	20	0	18	0	9	2	partial
13_1_40CM_Armatimonadetes_64_14	2092994	2092994		63.91	14.22	95	2133	67558	45	2	45	0	12	0	near
13_1_40CM_Actinobacteria_66_12	1591320	1591320		65.81	11.65	85	1721	57655	22	1	18	0	4	1	partial
13_1_40CM_Chloroflexi_68_21	1248088	1248088		67.5	21.51	90	1402	46524	17	0	17	0	6	0	partial
13_1_40CM_Gemmatimonadetes_70_12	495586	495586		69.57	11.54	43	527	21440	27	0	20	0	4	0	partial
13_1_40CM_Nitrospirae_62_7	865475	865475		61.97	7.04	76	929	18902	17	1	14	0	4	0	partial
13_1_40CM_Chloroflexi_55_7	967177	967177		55.21	6.65	87	1006	23530	25	0	22	0	3	0	partial
13_1_40CM_Archaea_48_12	790128	790128		48.4	12.14	52	951	49300	24	0	14	1	20	1	partial
13_1_40CM_Deltaproteobacteria_68_24	1175261	1175261		67.95	24.2	95	1208	24786	20	0	17	1	5	0	partial
13_1_40CM_Chloroflexi_67_9	946129	946129		67.19	9.08	73	1073	29577	16	0	14	1	5	1	partial
13_1_40CM_Chloroflexi_65_17	2627384	2627384		65.17	17.27	106	2780	115008	46	2	49	0	10	0	near
13_1_40CM_Gemmatimonadetes_66_11	2941586	2941586		65.9	10.69	205	3016	51267	5	1	11	1	8	2	partial
13_1_40CM_Gemmatimonadetes_70_11	1648210	1648210		69.9	11.41	123	1632	32605	15	3	16	3	7	0	partial
13_1_40CM_Acidobacteria_65_14	6386498	6386498		64.7	14.1	342	5678	79305	48	2	42	0	12	0	near
13_1_40CM_Chloroflexi_68_15	476670	476670		68.08	15.24	45	553	23296	19	0	18	0	3	0	partial
13_1_40CM_Gemmatimonadetes_69_22_partial	2363812	2363812		69.46	22.3	60	2213	156126	16	0	13	0	7	0	partial
13_1_40CM_Gemmatimonadetes_70_15_partial	1619003	1619003		69.62	15.47	104	1673	44923	23	0	25	0	9	0	partial
Sum	6.19E+08	45743342	7%												

Count	24	22	92%												

Four days after first rain plot 1 30 - 40 cm

Organism name	bin length	Partial to near complete	Bins/Megabins	GC%	coverage	# contigs	# features	longest contig	RP Inventory (total: 55)	RP multiple	BSCG Inventory (total: 51)	BSCG multiple	ASCG Inventory (total: 38)	ASCG multiple	completion status
13_1_40cm_2_UNK	1.43E+08			59.61	6.35	67659	216865	114328	54	51	51	49	38	38	megabin
13_1_40CM_2_Rokubacteria_70_45	2114328			69.87	45.08	328	2343	35736	41	1	39	0	11	0	partial
13_1_40CM_2_Myxococcales_68_15	1265159			67.89	14.91	465	1445	10091	25	1	22	0	5	1	partial
13_1_40CM_2_Nitrospirae_megabin_61_8	5823452			60.78	8.87	3435	7760	11081	46	32	46	34	14	9	megabin
13_1_40CM_2_Nitrospirae_62_10	2494038	2494038		61.7	10.15	657	2914	77373	40	7	39	0	11	4	partial
13_1_40CM_2_Deltaproteobacteria_megabin_63_4	49750827			63.14	4.07	27770	62182	17117	53	53	51	51	18	15	megabin
13_1_40CM_2_Actinobacteria_megabin_67_3	35105913			66.66	3.6	21490	47679	15721	52	51	51	50	17	16	megabin
13_1_40CM_2_Betaproteobacteria_megabin_64_4	27382188			64.07	4.14	16233	37139	14544	53	51	51	48	15	15	megabin
13_1_40CM_2_Acidobacteria_megabin_59_4	68929204			58.87	4.09	33803	79338	42138	53	53	51	51	17	15	megabin
13_1_40CM_2_Gemmatimonadetes_megabin_68_8	28624920			68.02	8.43	12303	34511	95148	53	53	51	51	16	15	megabin
13_1_40CM_2_Methylomirabilis_megabin_69_6	36084836			68.55	6.09	16527	48026	27622	53	51	51	49	15	13	megabin
13_1_40CM_2_Alphaproteobacteria_megabin_64_4	27267784			63.69	3.84	16831	36203	19637	52	48	47	40	14	9	megabin
13_1_40CM_2_Proteobacteria_megabin_63_4	34476873			63.35	4.77	15720	46249	27902	33	20	25	14	14	8	partial
13_1_40CM_2_Chloroflexi_megabin_53_3	13809904			53.15	2.7	8294	17611	14175	52	43	47	30	16	11	megabin
13_1_40CM_2_Chloroflexi_megabin_67_4	19441263			67.25	4.16	11131	24283	17754	51	48	50	47	17	14	megabin
13_1_40CM_2_Firmicutes_megabin_63_4	14433441			62.96	4.69	9090	18208	10994	46	41	45	40	17	14	megabin
13_1_40CM_2_Ignavibacteria_61_4	3851097			60.3	4.11	2044	4436	13884	39	8	33	4	9	4	partial
13_1_40CM_2_Verrucomicrobia_megabin_58_3	19328503			57.95	3	10931	24193	17562	51	50	50	48	16	16	megabin
13_1_40CM_2_GAL15_megabin_65_7	6882473			65.21	7.03	3817	9352	10836	46	41	45	36	14	9	megabin
13_1_40CM_2_Cyanobacteria_61_4	10911052			60.78	3.89	6823	12402	12046	7	2	3	0	6	1	partial
13_1_40CM_2_NC10_megabin_66_5	9464986			66.36	5.93	5393	12026	14113	21	13	20	16	11	7	partial
13_1_40CM_2_Thaumarchaeota_megabin_50_6	6701666			50.23	5.59	3512	8650	22785	33	27	22	12	37	34	megabin
13_1_40CM_2_Euryarchaeota_megabin_60_9	15067302			60.66	9.74	7783	18987	18991	44	39	23	21	38	37	megabin
13_1_40CM_2_Archaea_megabin_55_8	12657859			54.73	7.85	4210	15908	103008	40	38	23	20	37	37	megabin
13_1_40CM_2_remainingbacteria_63_5	1.95E+08			63.28	5.34	71600	259573	62572	53	53	51	51	23	17	megabin
13_1_40CM_2_Methylomirabilis_69_5	3726065	3726065		69.21	4.93	211	3960	69652	37	3	33	0	11	1	partial
13_1_40CM_2_Novel_65_8	2449862	2449862		65.04	7.98	127	2628	70218	19	1	21	1	6	0	partial
13_1_40CM_2_Gemmatimonadetes_69_13	3942005			69.21	13.15	197	3836	110443	45	27	43	24	11	5	megabin
13_1_40CM_2_Chloroflexi_67_6	138770	138770		66.57	5.87	13	161	14111	16	0	14	0	0	0	partial
13_1_40CM_2_Acidobacteria_56_11	3224912	3224912		55.67	11	222	3173	57034	24	1	26	0	10	0	partial
13_1_40CM_2_Archaea_52_13	1488827	1488827		51.89	12.98	78	1602	93184	19	4	6	0	17	1	partial
13_1_40CM_2_Novel_68_5	3168729	3168729		68.18	4.92	212	2905	71726	18	1	16	0	6	0	partial
13_1_40CM_2_Chloroflexi_68_14	3059620	3059620		68.07	14.18	209	3129	97911	42	6	42	5	7	2	near
13_1_40CM_2_Methylomirabilis_68_13	2347978	2347978		68.08	13.24	183	2583	52717	17	0	16	0	9	0	partial
13_1_40CM_2_Arch_MCG1_52_14	1696268	1696268		51.74	13.93	68	1812	66664	30	3	14	0	31	1	near
13_1_40CM_2_Archaea_52_4	826954	826954		51.88	4.25	65	910	43421	9	0	2	0	12	1	partial
13_1_40CM_2_Acidobacteria_68_10	1923720	1923720		67.9	9.85	122	1819	67439	42	2	39	0	9	0	partial
13_1_40CM_2_Acidobacteria_60_7	1983623	1983623		60.23	7.18	151	1798	54929	38	0	38	0	10	1	partial
13_1_40CM_2_Acidobacteria_56_5	1151268	1151268		56.45	5.29	101	1131	31695	23	0	21	0	11	0	partial
13_1_40CM_2_Gemmatimonadetes_70_7	1657495	1657495		69.92	6.73	104	1687	62952	23	0	21	0	10	0	partial
13_1_40CM_2_Novel_66_13	1142852	1142852		65.79	13.54	83	1233	30399	11	0	13	0	9	0	partial
13_1_40CM_2_Rokubacteria_68_8	2712366	2712366		68.41	7.93	184	2841	47280	32	4	31	1	9	1	partial
13_1_40CM_2_Novel_70_6	1662566	1662566		70.3	6.27	112	1828	55037	27	0	25	0	7	0	partial
13_1_40CM_2_Archaea_SAGMCG_39_13	555171			38.94	13.39	79	717	37220	24	2	9	0	20	0	partial
13_1_40CM_2_Archaea_39_4	901546			38.28	5.39	216	1108	40962	7	1	3	0	18	1	partial
13_1_40CM_2_Thaumarchaeota_39_13	804578			39.12	13.61	124	956	33336	7	0	4	0	18	1	partial
13_1_40CM_2_Thaumarchaeota_SAGMCG_39_7	1042608			38.64	6.6	216	1296	65502	29	3	11	0	25	1	partial
13_1_40CM_2_Gemmatimonadetes_60_3	1970774			59.98	2.82	1121	2468	8804	41	3	33	3	8	2	partial
13_1_40CM_2_CNBR_64_6	1458532			64.29	6.23	411	1516	35568	45	8	37	5	9	2	partial
Sum	8.35E+08	36855913	4%												
Count	49	18	37%												

Six days after first rain plot 1 30 - 40 cm

Organism name	bin length	Partial to near complete	Bins/Megabins	GC%	coverage	# contigs	# features	longest contig	RP Inventory (total: 55)	RP multiple	BSCG Inventory (total: 51)	BSCG multiple	ASCG Inventory (total: 38)	ASCG multiple	completion status
13_1_40cm_3_UNK	3.27E+08			61.73	5.8	135383	461827	98346	54	54	51	51	38	37	megabin
13_1_40CM_3_Nitrospirae_megabin_61_8	7780444			60.64	8.32	3888	10306	16887	49	38	46	31	13	11	megabin
13_1_40CM_3_Deltaproteobacteria_megabin_63_4	45996044			63.41	4.38	24271	57282	48143	53	53	51	51	17	16	megabin
13_1_40CM_3_Betaproteobacteria_megabin_64_4	30007082			64.33	4.46	16713	40253	17623	52	51	51	50	14	14	megabin
13_1_40CM_3_Alphaproteobacteria_megabin_63_4	26032200			63.59	3.97	15557	34635	17217	52	40	47	31	12	11	megabin
13_1_40CM_3_Acidobacteria_megabin_59_4	72149664			59.07	4.1	34109	83617	57326	53	53	51	51	16	16	megabin
13_1_40CM_3_Gemmatimonadetes_megabin_68_7	26328054			67.61	7.58	11289	32036	198509	53	53	51	50	15	15	megabin
13_1_40CM_3_Gammaproteobacteria_megabin_63_4	14332085			63.45	3.76	8742	17695	12592	46	21		43	17	14	megabin
13_1_40CM_3_Methylomirabilis_megabin_69_6	36264118			68.51	6.16	15508	48520	74444	52	50	49	48	16	15	megabin
13_1_40CM_3_Actinobacteria_megabin_67_4	32638971			66.71	3.84	19104	43987	31322	53	51	51	51	17	14	megabin
13_1_40CM_3_Proteobacteria_megabin_64_5	23469994			64.08	5.18	8011	33097	36505	26	13	20	9	8	3	partial
13_1_40CM_3_Chloroflexi_megabin_62_4	27175338			62.49	3.83	15538	34491	22365	53	52	50	50	17	13	megabin
13_1_40CM_3_Verrucomicrobia_megabin_59_3	7914524			59.16	3.07	5008	10025	12722	50	44	41	36	12	10	megabin
13_1_40CM_3_Verrucomicrobia_megabin_57_3	8612441			56.64	3.33	4631	11154	14853	49	47	47	45	15	14	megabin
13_1_40CM_3_Euryarchaeota_megabin_60_9	13928608			60.17	9.31	7444	18002	27367	40	38	28	20	38	37	megabin
13_1_40CM_3_Thaumarchaeota_megabin_48_6	6317791			47.72	6.12	3391	8219	15561	32	21	17	9	37	28	megabin
13_1_40CM_3_Archaea_megabin_53_8	11906279			53.15	7.91	4403	15468	71334	41	37	22	17	38	36	megabin
13_1_40CM_3_NC10_megabin_66_6	8950783			66.22	5.66	4971	11403	14366	22	13	21	10	11	9	partial
13_1_40CM_3_Firmicutes_megabin_63_5	12579550			62.87	4.55	7831	16129	12160	48	47	48	46	16	13	megabin
13_1_40CM_3_GAL15_megabin_65_7	6167310			65.02	6.79	3273	8369	12975	50	45	48	40	15	10	megabin
13_1_40CM_3_Delftia_acidovorans_66_6	4671791	4671791		66.29	6.28	292	4559	60339	47	1	44	0	10	0	near
13_1_40CM_3_Acidobacteria_55_5	2966753	2966753		55.17	4.51	176	2984	69897	16	1	14	0	8	1	partial
13_1_40CM_3_Gemmatimonadetes_66_12	2677766	2677766		65.73	12.16	128	2729	80773	19	0	21	0	10	1	partial
13_1_40CM_3_Novel_66_19	1137225	1137225		65.6	19.49	84	1259	33927	10	0	12	0	6	1	partial
13_1_40CM_3_Gemmatimonadetes_70_8	720177	720177		69.53	8.2	65	747	21671	8	0	9	0	5	0	partial
13_1_40CM_3_Gemmatimonadetes_65_8	1964318	1964318		64.83	7.94	119	2022	40766	13	0	11	0	5	1	partial
13_1_40CM_3_Deltaproteobacteria_69_14	2823300	2823300		68.63	13.89	148	2832	70931	20	1	19	1	6	0	partial
13_1_40CM_3_Acidobacteria_56_11	3419761	3419761		55.53	11.36	228	3488	44032	42	4	42	2	12	0	near
13_1_40CM_3_SAGMCG_38_6	1473591	1473591		37.9	6.47	86	1738	54535	30	2	11	1	31	3	near
13_1_40CM_3_Arch_MCG2_52_10	1345136	1345136		51.85	9.94	74	1492	54938	26	2	8	0	27	0	partial
13_1_40CM_3_Deltaproteobacteria_54_4	2236838	2236838		54.49	3.94	179	2398	31684	32	3	27	0	6	0	partial

13_1_40CM_3_Archaea_MCG_52_4	775972	775972		52.05	4.03	59	867	36390	23	3	5	0	14	0	partial
13_1_40CM_3_Nitrospirae_62_11	1100264	1100264		61.85	11.3	79	1152	29218	31	0	28	0	8	0	partial
13_1_40CM_3_Arch_MCG1_52_17	1771063	1771063		51.68	16.96	66	1902	127109	25	4	12	0	30	1	partial
13_1_40CM_3_Archaea_Thermoplasmatales_66_7	2325190	2325190		66.42	6.69	157	2433	50599	29	18	10	1	25	19	partial
13_1_40CM_3_Gemmatimonadetes_69_22	1685858	1685858		69.09	21.51	108	1762	42993	19	1	16	0	8	0	partial
13_1_40CM_3_Gemmatimonadetes_70_6	1589252	1589252		69.79	6.26	93	1575	51291	14	1	14	0	7	0	partial
13_1_40CM_3_Acidobacteria_65_5	3794804	3794804		65.06	4.65	304	3681	40292	24	0	21	0	5	0	partial
13_1_40CM_3_Chloroflexi_65_12	2380159	2380159		65.19	12.11	110	2574	69353	33	3	34	0	9	0	partial
13_1_40CM_3_Deltaproteobacteria_71_4	352886	352886		70.94	4.38	35	393	22504	25	0	24	0	2	0	partial
13_1_40CM_3_Acidobacteria_55_6	2366586	2366586		54.6	6.07	184	2326	37570	23	1	21	0	3	0	partial
13_1_40CM_3_Rokubacteria_69_38	2707031	2707031		69.53	37.81	362	3036	44365	42	3	41	0	11	2	near
13_1_40CM_3_Novel_70_6	559323	559323		69.7	6.22	49	636	23340	16	0	16	0	3	0	partial
13_1_40CM_3_Arch_MCG3_53_5	2497406	2497406		52.84	5.35	88	2581	273886	34	12	16	2	35	3	near
13_1_40CM_3_Arch_SCG1_50_5	1626692			49.56	5.53	370	2046	62769	30	2	17	1	35	3	near
13_1_40CM_3_Firmicutes_65_11	398295			65.39	11.43	186	517	8276	28	5	29	5	6	1	partial
Sum	7.97E+08	49342450	6%												
Count	46	24	52%												

2 days after second rain plot 1 30 - 40 cm

Organism name	bin length	Partial to near complete	Bins/Megabins	GC%	coverage	# contigs	# features	longest contig	RP Inventory (total: 55)	RP multiple	BSCG Inventory (total: 51)	BSCG multiple	ASCG Inventory (total: 38)	ASCG multiple	completion status
13_1_40cm_4_UNK	3.51E+08			62.07	5.82	133717	484839	105142	54	53	51	51	37	36	megabin
13_1_40CM_4_Nitrospirae_megabin_61_9	6042971			61.04	10.6	3234	7875	11659	47	35	44	27	13	11	megabin
13_1_40CM_4_Gemmatimonadetes_megabin_71_29	990116			70.5	28.81	429	1258	9774	37	13	32	7	6	1	partial
13_1_40CM_4_Deltaproteobacteria_megabin_68_18	1135657			67.75	17.56	488	1362	8774	20	4	19	2	6	1	partial
13_1_40CM_4_Methylomirabilis_megabin_68_6	26722786			68.01	6.84	11752	35806	61687	53	51	51	48	16	14	megabin
13_1_40CM_4_Deltaproteobacteria_megabin_63_4	39773945			63.35	4.4	21524	50604	22374	53	52	51	50	15	15	megabin
13_1_40CM_4_Euryarchaeota_megabin_60_10	13513956			60.02	10.44	6756	17102	30508	41	38	25	21	38	37	megabin
13_1_40CM_4_Thaumarchaeota_megabin_47_7	7325378			46.94	6.93	3666	9321	18666	33	30	17	11	36	33	megabin
13_1_40CM_4_Archaea_megabin_53_8	13838628			52.9	8.71	4580	17548	107010	39	36	23	17	38	38	megabin
13_1_40CM_4_Betaproteobacteria_megabin_64_5	22975250			64.04	4.75	12221	30853	18989	52	51	49	47	17	14	megabin
13_1_40CM_4_Alphaproteobacteria_megabin_63_4	22183612			63.51	4.25	13090	29656	15338	51	42	47	30	13	6	megabin
13_1_40CM_4_Proteobacteria_megabin_63_5	30948019			63.51	5.27	12845	41405	29121	26	16	24	15	14	7	partial
13_1_40CM_4_Acidobacteria_megabin_59_4	48644563			59.07	4.14	24467	57354	24222	53	53	51	51	17	14	megabin
13_1_40CM_4_Gemmatimonadetes_megabin_67_6	25359250			67.36	6.39	9501	29962	41164	53	53	50	50	14	14	megabin
13_1_40CM_4_Chloroflexi_megabin_67_5	17976645			66.76	5.06	9336	22315	24598	52	48	49	48	15	13	megabin
13_1_40CM_4_GAL15_megabin_65_7	6630185			64.75	6.97	3567	9093	10951	48	39	47	33	12	7	megabin
13_1_40CM_4_Chloroflexi_megabin_52_3	11429024			52.46	3.25	6253	14403	16950	53	42	45	38	14	13	megabin
13_1_40CM_4_Actinobacteria_megabin_66_4	30890358			66.24	4.24	16924	41333	26633	53	52	51	51	18	15	megabin
13_1_40CM_4_Verrucomicrobia_megabin_58_3	13591800			57.15	3.57	6918	17060	26719	50	49	47	46	16	15	megabin
13_1_40CM_4_Firmicutes_megabin_63_5	12583567			62.97	5.09	7463	15806	21644	52	48	48	45	17	14	megabin
13_1_40CM_4_Acidobacteria_57_6	1156824	1156824		57.09	5.85	98	1128	31165	14	0	12	0	2	0	partial
13_1_40CM_4_Chloroflexi_69_19	530753	530753		68.88	19.46	48	606	28183	7	0	8	0	4	0	partial
13_1_40CM_4_Gemmatimonadetes_69_5	1233548	1233548		69.4	4.85	92	1310	91978	20	0	16	0	8	0	partial
13_1_40CM_4_Novel_65_12	2726322	2726322		64.94	12.15	140	2862	73959	18	3	18	1	6	0	partial
13_1_40CM_4_Acidobacteria_56_10	4205805	4205805		55.6	10.24	241	4121	68800	44	0	44	0	13	0	near
13_1_40CM_4_Arch_SCG_48_7	1188454	1188454		48.38	7.36	70	1397	57055	26	0	16	1	28	0	partial
13_1_40CM_4_Acidobacteria_65_8	5226415	5226415		64.74	7.7	311	4667	74555	41	3	36	2	11	1	partial
13_1_40CM_4_Archaea_53_4	1625991	1625991		52.89	4.13	91	1784	75806	27	7	9	1	19	0	partial
13_1_40CM_4_Nitrospirae_62_6	1807939	1807939		61.5	6.31	108	1855	47416	43	0	41	0	10	0	near
13_1_40CM_4_Gemmatimonadetes_65_7	2627923	2627923		64.75	6.99	187	2711	56521	19	6	17	4	7	2	partial
13_1_40CM_4_Rokubacteria_67_11	3898620	3898620		67.46	10.55	279	4212	50176	24	7	23	2	10	2	partial
13_1_40CM_4_Rokubacteria_69_39	2937267	2937267		69.31	38.59	356	3190	48727	42	2	40	0	11	0	partial
13_1_40CM_4_Gemmatimonadetes_69_8	6148271			69.07	8.46	252	5883	123742	53	28	48	24	12	9	megabin
13_1_40CM_4_Betaproteobacteria_64_4	1644455	1644455		64.63	4.4	136	1758	34171	28	1	27	1	4	0	partial
13_1_40CM_4_Chloroflexi_65_16	1794243	1794243		65.28	16.01	121	1924	44587	38	1	41	1	10	0	near
13_1_40CM_4_Rokubacteria_69_5	4229972	4229972		69.01	5.17	189	4483	111011	46	4	41	0	13	0	near
13_1_40CM_4_Chloroflexi_65_13	499411	499411		65.04	12.75	22	537	68695	27	0	28	0	3	0	partial
13_1_40CM_4_Deltaproteobacteria_68_19	3477774	3477774		68.27	18.75	196	3509	75130	40	2	38	2	7	0	partial
13_1_40CM_4_Arch_SAGMCG_38_7	1402295	1402295		38.15	6.65	76	1634	46615	29	1	11	0	30	1	partial
13_1_40CM_4_Chloroflexi_68_4	3428701	3428701		67.67	4.42	203	3805	72344	42	3	39	3	9	2	partial
13_1_40CM_4_Acidobacteria_58_4	5659429	5659429		57.5	4.24	349	5490	79906	37	12	38	11	11	6	partial
13_1_40CM_4_Deltaproteobacteria_54_4	1210571	1210571		53.97	3.75	99	1319	34299	26	0	25	0	6	0	partial
13_1_40CM_4_Acidobacteria_61_5	1635232	1635232		60.85	5.06	125	1580	31777	26	0	31	0	10	0	partial
13_1_40CM_4_Chloroflexi_52_4	1439464	1439464		51.85	3.84	124	1439	31027	20	0	23	1	3	2	partial
13_1_40CM_4_Rokubacteria_71_6	4787378	4787378		70.85	6.09	299	5318	56297	19	9	20	5	9	2	partial
13_1_40CM_4_Verrucomicrobia_54_4	876475	876475		54.4	4.7	61	953	35517	22	0	18	0	6	0	partial
13_1_40CM_4_Novel_69_4	1464472	1464472		68.8	3.96	116	1385	25996	16	0	19	0	7	0	partial
Sum	7.72E+08	62715733	8%												
Count	47	26	55%												

Total for project	bin length	Partial to near complete	Bins/Megabins
Sum	6.7E+09	4.18E+08	6%
Count	372	180	48%
Average size of genomes (bp)		2.32E+06	

Table S3b. Genomes recovered from soil, NCBI, and JGI-IMG databases by phylum

	(10/7/2015 Searched	(10/7/2015 Genome Browser)	This work
Actinobacteria	991	3755	2

Acidobacteria	24	26	8
Proteobacteria	2392	12570	3
Nitrospirae	15	14	3
Rokubacteria	(BioSam ple)	0	7
Gemmatimonadetes	6	4	2
Chloroflexi	45	63	3
Verrucomicrobia	39	45	2
Armatimonadetes	3	4	1
Crenarchaeota	69	138	4
Thaumarchaeota	52	40	5
Total	3636	16659	40
