# Supplementary material for: Proteogenomic analyses indicate bacterial methylotrophy and archaeal heterotrophy are prevalent below the grass root zone
Source: PeerJ. 2016 Nov 8;4:e2687. doi: 10.7717/peerj.2687 (PMC5103831; doi:10.7717/peerj.2687)
Supplement: Table S4 — 2013 annual abundance survey and summary of plant species in the Angelo Coast Range Reserve meadow. Introduced species of summer flowering annual grasses and forbs were most abundant during this study. [file peerj-04-2687-s009.pdf]

Genus and Species	Common Name	Origin	Life History	Flowering Seasonality	2013 Abundance
(Eremocarpus) Croton setigerus	Turkey Mullein	Native	Annual Forb	Late Summer	High
(Linanthus) Leptosiphon bicolor	Linanthus, True Babystars	Native	Annual Forb	Spring	Low
**Achillea millefolium**	**Yarrow**	**Native**	**Perennial Forb**	**Late Summer**	**Southern margin only**
**Aira caryophyllea**	**Shiver grass**	**Introduced**	**Annual Grass**	**Early Summer**	**Low**
**Aphanes occidentalis**	**Lady's Mantle**	**Native**	**Annual Forb**	**Spring**	**Moderate**
Avena barbata	Slender wild oats	Introduced	Annual Grass	Early Summer	Trace
Briza minor	Little quakinggrass	Introduced	Annual Grass	Early Summer	Low
**Broadiaea elegans**	**Elegant Clusterlily**	**Native**	**Bulb (Liliaceae)**	**Early Summer**	**Moderate**
Bromus carinatus	California brome	Native	Annual/Short-lived	Early Summer	Trace
**Bromus diandrus**	**Ripgut grass**	**Introduced**	**Annual Grass**	**Early Summer**	**Moderate**
**Bromus hordeaceus**	**Soft Chess**	**Introduced**	**Annual Grass**	**Early Summer**	**Very High**
Bromus madritensis	Red Brome, Foxtail Chess	Introduced	Annual Grass	Early Summer	Trace
Bromus tectorum	Cheatgrass	Introduced	Annual Grass	Early Summer	Low
Cardamine oligosperma	Bitter-cress	Native	Annual Forb	Spring	Trace
Castilleja attenuata	Owl Clover	Native	Annual Forb	Early Summer	Low
Cerastium glomeratum	Mouse-ear Chickweed	Introduced	Annual Forb	Early Summer	Low
Cirsium occidentale	Cobwebby Thistle	Native	Perennial Forb	Early Summer	Trace
**Clarkia purpurea**		**Native**	**Annual Forb**	**Spring**	**Moderate**
Convulvus arvensis	Bindweed, Morning glory	Introduced	Perennial Forb	Summer	Trace
Cynosurus echinatus	Hedgehog dogtail	Introduced	Annual Grass	Summer	Moderate
**Danthonia californica**	**California oatgrass**	**Native**	**Perennial Grass**	**Summer**	**High**
**Daucus pusillus**	**Wild Carrot**	**Native**	**Annual or Biennial Forb**	**Summer**	**Moderate**
**Dichelostemma capitatum**	**Blue Dicks**	**Native**	**Bulb (Liliaceae)**	**Early Summer**	**Moderate**
Draba verna	Spring Draba	Native	Annual Forb	Spring	Trace
**Elymus glaucus**	**Blue wildrye**	**Native**	**Perennial Grass**	**Summer**	**Southern margin only**
**Elymus multisetus**	**Big Squirreltail grass**	**Native**	**Perennial Grass**	**Summer**	**Low**
Epilobium brachycarpum		Native	Annual Forb	Late Summer	Low
**Erodium cicutarium**	**Redstem Filaree, storksbill**	**Introduced**	**Annual Forb**	**Early Spring**	**High**
**Eschscholzia californica**	**California poppy**	**Native**	**Perennial Forb**	**All year**	**High**
**Gallium parisiense**	**Wall bedstraw**	**Introduced**	**Annual Forb**	**Early summer**	**Very High**
Gastridium ventricosum	Nitgrass	Introduced	Annual Grass	Early Summer	Trace
Geranium dissectum	storks bill	Introduced	Annual Forb	Spring	Low
Hemizonia congesta	Hayfield tarweed	Native	Annual Forb	Summer	Trace
Hypochaeris glabra	Smooth Cat's Ear	Introduced	Annual Forb	Summer	Low
**Lotus micranthus**		**Native**	**N-fixer**	**Early Summer**	**Very High**
Lotus wrangelianus	Yellow Lotus	Native	N-fixer	Early Summer	Low
**Lupinus bicolor**	**Annual Lupine**	**Native**	**Annual Forb**	**Early Summer**	**High**
Madia gracilis	Slender Tarweed	Native	Annual Forb	Mid Summer	Low
Micropos californicus	Slender cottonweed	Native	Annual Forb	Early Summer	Trace
Myosotis discolor	Changing Forget-me-not	Introduced	Annual/Biennial Forb	Spring	Trace
Navarretia divaricata	Mountain Navarretia	Native	Annual Forb	Spring	Trace
**Plagiobothrys nothofulvus**	**Popcorn flower**	**Native**	**Annual Forb**	**Spring**	**Moderate**
Platystemon californicus	Cream cups	Native	Annual Forb	Early Summer	Trace
Ranunculus occidentalis	Western Buttercup	Native	Perennial Forb	Spring	Trace
**Rumex acetosella**	**Sheep sorrel**	**Introduced**	**Perennial Forb**	**All year**	**High**
**Sanicula bipinnatifida**	**Purple sanicle**	**Native**	**Perennial Forb**	**All year**	**High**
**Sherardia arvensis**			**Annual Forb**	**Early Spring**	**High**
Stachys ajugoides		Native	Perennial Forb	Mid Summer	Trace
Torilis arvensis	Field Hedge Parsley	Introduced	Annual Forb	Mid Summer	Low
Trichostema lanceolatum	Vinegarweed	Native	Annual Forb	Late Summer	Very High
**Trifolium albopurpureum**		**Native**	**Annual N-fixer**	**Early Summer**	**Moderate**
Trifolium bifidum		Native	Annual N-fixer	Early Summer	Low
**Trifolium microcephalum**		**Native**	**Annual N-fixer**	**Early Summer**	**Moderate**
Trifolium willdenovii		Native	Annual N-fixer	Early Summer	Low
**Vulpia myuros**		**Introduced**	**Annual Grass**	**Early Summer**	**Very High**

Particularly abundant at site within meadow:

(green) **= Flowering end of August, secescing into September and October**

(yellow) **= Dead and senescing tissues August through October**
