# Supplementary material for: Proteogenomic analyses indicate bacterial methylotrophy and archaeal heterotrophy are prevalent below the grass root zone
Source: PeerJ. 2016 Nov 8;4:e2687. doi: 10.7717/peerj.2687 (PMC5103831; doi:10.7717/peerj.2687)
Supplement: Table S5 — Chemical formula and neutral mass for each compound in Figure 5 is listed along with LC-MS retention times and the ions associated with that metabolite. The primary ion used for analysis of the metabolite abundance is listed (selected based on detection limits and coefficient of variation across sample extractions) along with secondary ions and primary MS/MS fragments where available. For each ion entry the polarity (POS–positive ionization mode vs NEG–negative ionization mode), adduct (e.g., H+, H-, Na+) and m/z value are presented. Internal extraction and resuspension standards used are listed at the bottom of the table. [file peerj-04-2687-s010.pdf]

Label[1]	Neutral Mass	Formula[2]	Sample Retention Time (min)	Standard[3]	Standard Retention Time	Ion (m/z) used for Sample Analyses[4]	Additional ions[5]	Largest MS/MS fragments[6]
C5-Disaccharide (xylobiose) 1	282.0951	C10H18O9	11.4	Xylobiose	11.3	[NEG, H-] 281.0878	[POS, Na+] 305.0841; [NEG, Cl-] 317.0641	
C5-Disaccharide (xylobiose) 2	282.0951	C10H18O9	10.8	Xylobiose	10.7	[NEG, H-] 281.0878	[POS, Na+] 305.0841; [NEG, Cl-] 317.0641	
C12H22O10 (Rutinose) 1	326.1213	C12H22O10	12	Rutinose	11.7,12.1	[NEG, H-] 325.114	[POS, Na+] 349.1103	[NEG, 325.114 @ 20V] 163.0610, 103.0404; [POS, 349.1103 @ 20V] 149.0245
C12H22O10 (Rutinose) 2	326.1213	C12H22O10	11.6	Rutinose	11.7,12.1	[NEG, H-] 325.114	[POS, Na+] 349.1103	[NEG, 325.114 @ 20V] 163.0610, 103.0404; [POS, 349.1103 @ 20V] 149.0245
(C13H24O11)	356.1319	C13H24O11	10.8			[POS, NA+] 379.1198	[NEG, H-] 355.1246	[POS, 379.1121 @ 10V] 185.0417, 217.066
Sugar Alcohol 1 (Mannitol)	182.07904	C6H14O6	10.4	Mannitol	10.6	[NEG, H-] 181.0718	[POS, H+] 183.0863	[NEG, 181.073 @ 20V] 80.9166, 71.0136, 89.0235, 101.0241
Sugar Alcohol 2 (Xylitol)	152.06848	C5H12O5	8.8	Xylitol	8.9	[NEG, H-] 151.0613		[NEG, 151.063 @ 10V] 89.0231, 101.0234, 71.0147, 73.029
Sugar Alochol 3 (Unk C5)	152.06848	C5H12O5	7.9			[NEG, H-] 151.0613		
Hexose 1 (Glucose/Galactose)	180.06339	C6H12O6	10.2	13C6-Glucose ISTD	10.2	[NEG, H-] 179.0565	[NEG, Cl-] 215.0324, [NEG, +Hac-H] 239.0778	
Hexose 2 (Glucose/Galactose)	180.06339	C6H12O6	10.8	13C6-Glucose ISTD	10.8	[NEG, H-] 179.0565	[NEG, Cl-] 215.0324, [NEG, +Hac-H] 239.0778	
(Hexose 3)	180.06339	C6H12O6	12			[NEG, H-] 179.0565	[NEG, Cl-] 215.0324, [NEG, +Hac-H] 239.0778	
(Hexose 4)	180.06339	C6H12O6	13.1			[NEG, H-] 179.0565	[NEG, Cl-] 215.0324, [NEG, +Hac-H] 239.0778	
(Hexose 5)	180.06339	C6H12O6	9.7			[NEG, H-] 179.0565		
Hexose 6 (Fructose)	180.06339	C6H12O6	9.1	Fructose	9	[POS, Na+] 203.0531	[NEG, H-] 179.0565; [NEG, Cl-] 215.0324 ;	
Hexose 7 (Mannose)	180.06339	C6H12O6	10.1	Mannose	10.4	[POS, Na+] 203.0531	[NEG, +Hac-H] 239.0778	
Trehalose	342.11622	C12H22O11	13.8	D-(+)-Trehalose	13.8	[NEG,+Hac-H] 401.1302	[NEG, H-] 341.1096; [NEG, Cl-] 377.0852; [NEG, Fragment] 179.0565; [POS, Na+] 365.1062; [POS, NH4+] 360.1507	
Sucrose	342.11622	C12H22O11	12.6	Sucrose	12.7	[NEG, H-] 341.1096	[NEG, Cl-] 377.0852; [NEG,+Hac-H] 401.1302	
Disaccharide 3 (Cellobiose/Maltose)	342.11622	C12H22O11	13.4	Cellobiose/Maltose	13.2,13.4	[NEG, H-] 341.1096	[NEG,+Hac-H] 401.1302; [NEG, Cl-] 377.0852; [POS, Na+] 365.1062; [POS, NH4+] 360.1507	
(Disaccharide 6)	342.11622	C12H22O11	15.8			[POS, Na+] 365.1062	[POS, NH4+] 360.1490	
Trisaccharide 1	504.169	C18H32O16	15.5			[NEG, H-] 503.162	[NEG, Cl-] 539.138; [NEG, +Hac-H] 563.1828; [POS, Na+] 527.1583; [POS, NH4+] 522.203	[NEG, 503.158 @ 20 V] 341.1074, 89.0238, 221.0667, 119.0371, 179.0555
Trisaccharide 2	504.169	C18H32O16	15	D-(+)-Raffinose	15	[NEG, H-] 503.162	[NEG, Cl-] 539.138; [NEG, +Hac-H] 563.1828; [POS, Na+] 527.1583; [POS, NH4+] 522.203	
Trisaccharide 3	504.169	C18H32O16	14.3			[POS, Na+] 527.1583	[NEG, Cl-] 539.138; [NEG, +Hac-H] 563.1828, [NEG, H-] 503.162, [POS, NH4+] 522.203	[POS, 527.1583 @ 20V] 185.0407, 201.0367, 111.9523, 467.6755
Trisaccharide 4	504.169	C18H32O16	13.7			[POS, Na+] 527.1583	[NEG, Cl-] 539.138; [NEG, +Hac-H] 563.1828, [NEG, H-] 503.162, [POS, NH4+] 522.203	
Trisaccharide 5	504.169	C18H32O16	14.9			[NEG, H-] 503.162	[NEG, Cl-] 539.138; [NEG, +Hac-H] 563.1828; [POS, Na+] 527.1583; [POS, NH4+] 522.203	
Trisaccharide 6	504.169	C18H32O16	13.1			[POS, Na+] 527.1583	[NEG, Cl-] 539.138; [NEG, +Hac-H] 563.1828, [NEG, H-] 503.162, [POS, NH4+] 522.203	
Tetrasaccharide 1 (Maltotetraose)	666.22186	C24H42O21	16	Maltotetrose	16	[NEG,H-] 665.2145	[POS, Na+] 689.2109; [POS, NH4+] 684.256; [NEG, Cl-] 701.191; [NEG, +Hac-H] 725.2357	
Tetrasaccharide 2 (Stachyose)	666.22186	C24H42O21	16.9	Stachyose	16.9	[NEG,H-] 665.2145	[POS, Na+] 689.2109; [POS, NH4+] 684.256; [NEG, Cl-] 701.191; [NEG, +Hac-H] 725.2357	[NEG, 665.2145 @40V] 89.025, 383.1175, 221.0661, 179.0565; [POS, 689.2109 @ 40V] 527.1577, 365.1028
Tetrasaccharide 3	666.22186	C24H42O21	16.3			[NEG,H-] 665.2145	[POS, Na+] 689.2109; [POS, NH4+] 684.256; [NEG, +Hac-H] 725.2357	
Tetrasaccharide 4	666.22186	C24H42O21	14.9			[NEG,H-] 665.2145	[POS, Na+] 689.2109; [POS, NH4+] 684.256	
Tetrasaccharide 6	666.22186	C24H42O21	14.5			[POS, Na+] 689.2109	[POS, fragment] 527.1582 ;[NEG, H-] 665.2145; [POS, NH4+] 684.256; [NEG, Cl-] 701.191; [NEG, +Hac-H] 725.2357	
Pentasaccharide 1	828.2747	C30H52O26	16.8	Maltopentose	17	[NEG, H-] 827.2663	[POS, Na+] 851.2637; [NEG, Cl-] 863.2437; [NEG, +Hac-H] 887.2885	[NEG, 827.2663 @ 40V] 383.1167, 323.0973, 647.1953, 131.0314

Pentasaccharide 2	828.2747	C30H52O26	17.8			[NEG, H-] 827.2663	[POS, Na+] 851.2637	[NEG, 827.2663 @ 40V] 383.1168, 179.0580, 647.1998, 443.099
Pentasaccharide 3	828.2747	C30H52O26	15.5			[NEG, H-] 827.2663	[POS, Na+] 851.2637	
Hexasaccharide 1	990.3275	C36H62O31	17.3	Maltohexose	17.3	[POS, NH4+] 1008.3619	[NEG, H-] 989.3202; [POS, Na+] 1013.3165; [NEG, H-H20-] 971.3003	
Hexasaccharide 2	990.3275	C36H62O31	18.6			[POS, NH4+] 1008.3619	[NEG, H-] 989.3202; [POS, Na+] 1013.3165	
Tryptophan	204.08988	C11H12N2O2	9.3	L-Tryptophan, D-Tryptophan	9.4	[NEG, H-] 203.0826	[POS, H+] 205.0972	
L-Leucine	131.09463	C6H13NO2	8.8	L-Isoleucine	8.7	[NEG, H-] 130.0874	[POS, H+] 132.1022	
L-Isoleucine	131.09463	C6H13NO2	8.4	L-Leucine	8.4	[NEG, H-] 130.0874	[POS, H+] 132.1022	
Valine	117.07898	C5H11NO2	9.8	L-Valine	9.9	[NEG, H-] 116.0717	[POS, H+] 118.0864	
Phenylalanine	165.07898	C9H11NO2	8.4	L-Phenylalanine	8.5	[NEG, H-] 164.0717	[POS, H+] 166.0863	
(Sarcosine)	89.04768	C3H7NO2	11.5	Sarcosine	11.2	[POS, H+] 90.0551	[NEG, H-] 88.0403	
(N-acetyl-l-phenylalanine)	207.08954	C11H13NO3	4.4	N-Acetyl-L-Phenylalanine	4.5	[NEG, H-] 206.08226		
(4-aminobutanoate)	103.06333	C4H9NO2	12.6	4-Aminobutanoate	12.9	[POS, H+] 104.0706		
Proline	115.06333	C5H9NO2	9.4	L-Proline	9.3	[NEG, H-] 114.056	[POS, H+] 116.0707	
Tyrosine	181.07389	C9H11NO3	10.6	L-Tyrosine	10.5	[NEG, H-] 180.067	[POS, H+] 182.0813	
Deoxyguanosine	267.0968	C10H13N5O4	7.9	Deoxyguanosine	7.9	[POS, fragment] 151.0494	[POS, H+] 268.1031	
L-Carnitine	161.10519	C7H15NO3	10.4	L-Carnitine	10.5	[POS, H+] 162.1125	[POS, Na+] 184.0942	[POS 162.1125 @ 10V] 85.0244, 60.081, 65.025, 121.0465, 103.040
Acetylcarnitine	203.1158	C9H17NO4	7.8	O-Acetyl-l-carnitine	7.8	[POS, H+] 204.123	[POS, fragment] 162.113	[POS, 204.123 @ 10V] 85.0273, 145.0508
(C8H16N2O3)	188.11609	C8H16N2O3	8.6			[POS, H+] 189.1233		[POS, 189.1243 @ 10V] 130.0499, 85.0285, 60.0804
(N6-Acetyl-lysine)	188.11609	(C8H16N2O3)	10.4	N6-Acetyl-lysine	11	[POS, H+] 189.1233		[POS, 189.1243 @ 10V] 84.0797, 126.0903
Betaine	117.07898	C5H11NO2	7	betaine (glycine-betaine)	7.6	[POS, H+] 118.0864	[POS, NA+] 140.0682, [POS, fragment] 100.076	
(C8H16N2O3)2	188.11609	(C8H16N2O3)	9.5			[POS, H+] 189.1233		
Glutamine	146.06914	C5H10N2O3	13	L-Glutamine	13.2	[NEG, H-] 145.0618	[POS, H+] 147.0766	
Cytidine	265.067424	C9H13N3O5	8.2	Cytidine	8.2	[POS, H+] 266.0747	[POS, fragment] 112.0504	
Asparagine	132.1179	C4H8N2O3	13.1	L-Asparagine	13.2	[POS, H+] 133.1251		
Ectoine	142.0742	C6H10N2O2	9.6	Ectoine	9.9	[POS, H+] 143.0815		
Cytosine	111.04326	C4H5N3O	6.7	Cytosine	7.2	[POS, H+] 112.0505		
Deoxycytidine	227.09061	C9H13N3O4	6.6	Deoxycytidine	7.2	[POS, H+] 228.0979	[POS, fragment] 112.0504	
Adenine	135.0545	C5H5N5	5	Adenine	5.1	[POS, H+] 136.0618		
Inosine	268.08077	C10H12N4O5	7.9	Inosine	7.5	[POS, fragment] 137.0456		
Choline-O-Sulfate	183.056914	C5H13NO4S	6.1	Cholinesulfate	6.1	[POS, H+] 184.0642	[POS, fragment] 104.1076	[POS, 184.0642 @ 10V] 104.1073
Uridine	244.0695	C9H12N2O6	5.4	Uridine	5.6	[POS, H+] 245.0768	[NEG, H-] 243.0623	
Deoxyadenosine	251.1018	C10H13N5O3	4.2	2'-Deoxyadenosine	4.4	[POS, H+] 252.1095	[POS,fragment] 136.062	
Adenosine	267.09676	C10H13N5O4	5.1	Adenosine	5.3	[POS, H+] 268.1040		
Uracil	112.02728	C4H4N2O2	3.4	Uracil	3.8	[NEG, H-] 111.021		
Hypoxanthine	136.03851	C5H4N4O	5.7	Hypoxanthine	6.2	[POS, H+] 137.0458		
Deoxyuridine	228.07462	C9H12N2O5	3.6	Deoxyuridine	4	[NEG, H-] 227.0673	[NEG, fragment] 111.021	
Serine	105.0926	C3H7NO3	13.1	L-Serine	13.2	[NEG, H-] 104.036		
Guanosine	283.09167	C10H13N5O5	9.3	Guanosine	9.7	[POS, H+] 284.099		
(C6H11NO3)	145.0739	(C6H11NO3)	8.9			[POS, H+] 146.0812	[POS, fragment] 100.0756, [NEG, H-] 144.066	

Extraction Standards

Name	Neutral Mass	Formula	Retention Time (min)	Positive Ions	Negative Ions
ABMBA	228.9738	C8H8BrNO2	2.99	[POS, H+] 229.9811	[NEG, H-] 227.9666
UL-13C-Glucose	186.0814	[13C]6H12O6	10.2, 10.8	[POS, Na+] 209.0704	[NEG, H-] 185.25411
**Internal Standards**					
9-antracene carboxylic acid	222.068085	C15H10O2	3.11		[NEG, H-] 221.0608
d5-benzoic acid	127.068161	C7HD5O2	4.5		[NEG, H-] 126.0609

3,6-Dihydroxy-4-methylpyridazine	126.042931	C5H6N2O2	6.1	[POS, H+] 127.0503	[NEG, H-] 125.0357
4-(3,3-dimethyl-ureido)benzoic acid	208.084793	C10H12N2O3	6.7	[POS, H+] 209.0921	[NEG, H-] 207.0775
13C-15N-L-phenylalanine	175.106201	[13C]9H11[15N]O2	8.4	[POS, H+] 176.1135	[NEG, H-] 174.0989
d4 lysine	150.13063	C6H10D4N2O2	23.7	[POS, H+] 151.1379	[NEG, H-] 149.1233

1 - Corresponds to the label in the heat map
2 - Compound formulas are derived from compound if known, otherwise it was from a combination of isotope patterns, neutral losses, elution time and MS/MS if available
3 - Standards from the Northen Lab chemical library that were analyzed using the same column and gradient
4 - ions (m/z values) used for evaluating compounds across samples were selected based on their sensitivites and CV among replicate analyses
5 - Additional co-eluting ions that were used to confirm compound, neutral mass and type
6 - Selected MS/MS patterns observed on sample compounds that were present at sufficient abundance.
