# Supplementary material for: Proteogenomic analyses indicate bacterial methylotrophy and archaeal heterotrophy are prevalent below the grass root zone
Source: PeerJ. 2016 Nov 8;4:e2687. doi: 10.7717/peerj.2687 (PMC5103831; doi:10.7717/peerj.2687)
Supplement: Table S6 — Peak areas of each metabolite used to generate Figure 5 and analyzed for significant changes (Table S7). Peak areas correspond to the ‘Ion (m/z) used for Sample Analyses’ column in Table S5. [file peerj-04-2687-s011.pdf]

Table A. Sugar Compound Peak Areas Used to Generate Figure 5 (Upper)

compound	Cpd Group	Polarity	820_10_20A.mzML	820_10_20B.mzML	820_10_20C.mzML	925_10_20A.mzML	925_10_20B.mzML	925_10_20C.mzML	927p1_10_20A.mzML	927p1_10_20B.mzML	927p1_10_20C.mzML	1002p1_10_20A.mzML	1002p1_10_20B.mzML	1002p1_10_20C.mzML	820_30_40A.mzML	820_30_40B.mzML	820_30_40C.mzML	925_30_40A.mzML	925_30_40B.mzML	925_30_40C.mzML	927p1_30_40A.mzML	927p1_30_40B.mzML	927p1_30_40C.mzML	1002p1_30_40A.mzML	1002p1_30_40B.mzML	1002p1_30_40C.mzML	control1.mzML	control2.mzML	control3.mzML	control4.mzML
C5-Disaccharide (xylobiose) 1	C5 Disaccharide	NEG	47566.37	41296.78	91300.22	358318.3	284726.4	378355.5	83464.1	102200.6	95241.48	168120.1	2519.88	126619.4	11915.53	2892.8	5306.5	6105.53	4066.91	1200.57	7176.04	2409.27	0	11156.07	817.4	11749.7	0	0	0	0
C5-Disaccharide (xylobiose) 2	C5 Disaccharide	NEG	70743.04	59078.22	130956.1	437005.1	346342.8	448169.5	113514.8	128702.6	114270.1	233675.9	172254.6	100546.3	12319.72	4179.54	7416.81	960.28	4287.69	2132.78	7690.41	4071.46	0	19685.79	4401.58	23391.38	0	20	0	0
(Disaccharide6)	C6 Disaccharide	POS	116221.7	128507.7	123057.2	165790.7	87452.31	114222.6	102503.6	106791.2	132589.9	109406.5	180636.8	153007.2	7143.72	5707.81	6275.53	12100.67	7564.24	7079.8	5581.16	3888.96	4285.06	2563.98	2636.57	3268.29	0	0	87.01	0
(Hexose3)	C6 saccharide	NEG	1049401	1069831	1079400	903983.1	863432.5	957127.5	1077644	1234160	1197457	1922558	3145119	2720344	151046.3	120047.7	154777.5	111036	89749.16	94249.05	96521.07	88358.18	22747.4	29474.77	5355.04	31914.26	91.03	453.59	151.9	157.94
(Hexose4)	C6 saccharide	NEG	602635.1	597975	608176	523015.5	483091	514779.8	606626	750743	724386.2	1593670	2663065	2330112	117960.5	87418.16	123393.8	83346.32	64119.76	67422.15	80819.35	76773.45	71557.2	28998.82	4355.23	33403.9	58.34	504.12	248.58	272.9
(Hexose5)	C6 saccharide	NEG	63738356	56117715	60882667	55850317	67557417	67498593	50102653	57537549	55605958	1.85E+08	1.91E+08	1.71E+08	15219163	14068745	17043181	4990080	7683182	9513199	6850265	9123293	82192.49	2832486	2163619	4294859	261.21	46003.43	180.29	20217.42
Pentasaccharide1	C6 Pentasaccharide	NEG	546235	482801.4	551034.8	520544.7	424320.6	422228.5	508484.5	613238.6	569664.2	203944.3	444830	318204.8	95833.73	86050.66	108829.8	115859.1	122431.7	107241.9	99773.89	108004.5	91282.33	134030.7	102247.6	140725.8	0	0	0	0
Pentasaccharide2	C6 Pentasaccharide	NEG	53323.33	49639.67	55391.76	34736.4	29668.67	33640.3	38189.06	50942.44	48478.87	5820.85	22171.21	12566.87	136.73	368.58	67.63	242.64	615.4	363.54	0	0	81.99	0	0	0	0	0	0	0
Pentasaccharide3	C6 Pentasaccharide	NEG	550715.9	489960	557368.7	566694.1	532393.1	537072.5	510613.6	615379.1	572613.9	218450.2	452069.2	328251.1	95833.73	86050.66	110017.7	115859.1	122431.7	107241.9	99773.89	108004.5	91252.54	134030.7	102247.6	140725.8	0	0	0	0
Tetrasaccharide3	C6 Tetrasaccharide	NEG	326647.5	303700.4	351590.3	268575.6	197601.5	213383.3	272386.5	309598	280785.7	69762.5	173034.2	117898.2	24033.06	20955.13	26140.6	24912.4	25237.25	25847.71	16922.72	15870.05	13584.93	1673.52	13.87	1892	0	0	0	0
Tetrasaccharide4	C6 Tetrasaccharide	NEG	1555671	1485605	1636816	1407431	1019988	1068469	1427290	1788546	1662703	585062.5	1103102	891171.1	361334	308089.1	490530.9	403829.3	460970.5	382455.1	379182.9	311392.1	249692.6	471245.6	126433.7	456191.2	0	0	0	0
Tetrasaccharide6	C6 Tetrasaccharide	POS	1027982	1100735	1172539	1000742	845641.2	905775.6	1094160	1020828	1252563	515042	797440.6	619461.3	237348.6	244917.5	297116.9	300623.6	289493	294520.6	207388.2	225410.3	205003	367839.5	331486.9	351440.4	0	0	0	0
Trisaccharide1	C6 Trisaccharide	NEG	1119575	1052363	1395210	862233.7	632120.5	734464.8	774227.8	915380.6	768578.5	640296.2	1112965	914680	180577.9	122486	163862.2	105417.2	143015.5	151849.4	140475.7	102918.7	95936.29	49678.63	0	42301.52	0	327.63	0	232.74
Trisaccharide2	C6 Trisaccharide	NEG	3945570	4864214	4776383	3884795	3339932	3695521	3311267	3945381	3331295	2647629	4054595	3457704	1019794	706878	941223.5	652579.4	811516.6	713163	780004.5	699764.5	572595.7	525105.5	91616.84	472497.1	31351.34	32078.1	32585.9	33563.81
Trisaccharide3	C6 Trisaccharide	POS	4408065	4659119	4673476	4267063	3893001	4161572	4615398	4799222	5489869	3367190	4241666	3760800	1092804	1089275	1251689	1013533	980835.8	1019288	869663.8	925201	839109.1	804056.9	763784.7	752810.1	44.24	0	5901.92	0
Trisaccharide4	C6 Trisaccharide	POS	4612012	4910100	4849344	4386866	4049124	4305426	4751762	4846413	5517901	3465778	4456132	3976595	1106541	1102503	1354021	1093777	1073046	1106476	879603.3	932573.1	841684.4	812949.1	768814.7	754893.2	44.24	0	5901.92	0
Trisaccharide5	C6 Trisaccharide	NEG	4093789	5002863	4947692	4028397	3384134	3763651	3352155	4005113	3393049	2689177	4211474	3540734	1092429	768636.6	1237946	703801.9	865700	767110.8	821327.4	727448.9	604901.7	554497.4	96460.03	513374.4	31351.34	32078.1	32585.9	33549.84
Trisaccharide6	C6 Trisaccharide	POS	286916.8	324691.9	314218.7	135026.8	130728.6	150850.9	149742.3	132369.7	167624	127800.7	237408.5	235685.8	18650.55	13804.31	95091	36035.28	33645.54	34670.98	3080.83	3601.39	3936.7	458.34	716.95	613.22	0	0	0	0
Hexasaccharide1	C6 Hexasaccharide	POS	180545	190391	224652.7	196143.2	162553.3	175335.7	200990.5	185574.3	210686.6	90206.45	155179.1	104042.2	31259.91	32629.03	34747.1	57995.51	54704.93	51519.65	46216.43	46870.71	44547.27	54961.92	51475.77	55765.9	0	0	0	0
Hexasaccharide2	C6 Hexasaccharide	POS	4825.3	4941.57	5962.27	2679.73	2860.56	5149.73	3784.91	4239.17	5527.47	30.23	787.28	152.45	0	0	0	0	0	0	0	0	0	0	0	0	0	0	0	0
Trehalose	C6 Disaccharide	NEG	66642961	64310325	72755214	61954111	53090817	53357898	55862717	63669435	57796142	23630552	37148446	34361437	29559729	22750970	25962155	23998571	26975203	24264753	24316504	23754721	22031988	13954013	452423.2	12924024	10338.62	6807.41	7063.84	9089.79
Sucrose	C6 Disaccharide	NEG	48108278	49576165	48907019	34252228	34597812	36142844	31184369	33064803	32820027	12352517	17669645	15571279	23670006	17472722	25454205	23479922	24596541	23129022	10506883	12364131	9973768	4231250	1261974	4324252	1980.67	1307.79	1100.42	3851.18
Disaccharide3 (cellobiose/ maltose)	C6 Disaccharide	NEG	31295442	31358225	31773100	27831968	27224964	28839866	25547919	28112106	27728200	12099837	17233440	15035444	10996455	8074314	10702729	8697387	9262725	8263330	8000343	9562200	8185995	3791732	1038224	3559798	2004.76	96.31	1085.59	3847.51
Hexose1 (glucose/ galactose)	C6 saccharide	NEG	60033887	49160969	56970833	54336264	66048561	65853219	49420659	56662428	54856037	1.84E+08	1.89E+08	1.74E+08	14693295	13594866	16293943	8000369	7495601	9267182	6804077	9055657	5430757	2798890	1939319	4101730	14841.76	55676.1	7533.85	32067.08
Hexose2 (glucose/ galactose)	C6 saccharide	NEG	37963608	28266325	35147536	35578718	48698637	45358182	31298386	34013303	36793301	1.4E+08	1.58E+08	1.4E+08	9839242	8647593	10802555	4658745	5329572	5999974	4805514	6009885	3537077	1860047	1243136	2324487	11795.54	39269.4	5319.02	24317.56
Hexose6 (fructose)	C6 saccharide	POS	212721.3	241322.7	221254.7	107201.7	134351.6	126591.5	113751.2	94578.48	118139.9	177201.5	32601.99	192522.7	122805.4	112703.7	129657.1	154416.6	156764	150628.1	34807.86	38078.66	29672.52	4311.81	6595.37	11966.73	123.86	129.65	103.31	204.89
Hexose7 (mannose)	C6 saccharide	POS	23417364	25120468	21776362	17774004	21856181	22220156	17474868	18632588	25311643	1.08E+08	1.01E+08	1.29E+08	7215226	5574636	6404806	5731497	4823804	4560161	3906178	3866699	5267363	1016641	1205611	1971991	50489.67	52721.04	48257.96	54397.34
SugarAlc1 (mannitol)	Sugar alcohol	NEG	8554779	7745437	8473923	5742970	5529001	6023263	6874656	8706566	8309786	12062748	19610789	16862080	219165.1	181101.4	214017	218537.5	129649.6	183619.2	146271.3	129945.1	119646.4	49146.36	20882.35	39787.57	7591.25	1676.77	470.17	995.41
Tetrasaccharide1 (maltotetraose)	C6 Tetrasaccharide	NEG	1468592	1411613	1540696	1363172	978268.7	1017421	1372644	1719532	1602071	531186.5	1036230	821479.3	360188.3	307704.9	385983.2	393440.6	460498.9	382344.5	378519.6	311180.6	264113.2	471245.6	124209.6	456009.3	0	0	0	0
Tetrasaccharide2 (stachyose)	C6 Tetrasaccharide	NEG	461171.9	411993.3	483328.9	387906.5	268398.1	292992.1	410463.7	483465.2	445881.3	143773	328795.5	239260.4	38277.78	31726.23	34760.62	38703.23	38121.1	42449.73	25712.66	20967.17	18222.94	6813.24	13.87	9837.23	0	0	0	0
C12H22O10 (Rutinose)1	C6 Disaccharide other	NEG	65961.55	66170.45	82177.12	111163.8	124438.2	136212.3	204890.4	258989.1	184980.2	123785.3	175654.7	165748.4	63808.95	35560.75	38908.14	47729.98	73022.14	49186.91	37547.95	29296.58	99258	29780.83	11991.29	20082.74	0	0	0	0
C12H22O10 (Rutinose)2	C6 Disaccharide other	NEG	135288.4	132195.1	159191.7	266562.8	336262.1	345521.3	434955.6	501334.1	440964.3	360111.5	444877.3	386065.5	138862.8	88670.07	90614.82	76884	158565.1	112610.9	84380.62	68837.11	113073	39194.19	11991.29	32336.76	0	0	0	0
(C13H24O11)	C6 saccharide other	POS	3521698	3758753	3526035	1259242	1143793	1305705	2763003	2793067	3734941	12549369	16072366	16200387	392941.1	339256.6	364764.1	132724.5	129678.1	132620.6	102788.4	111140.4	102416	60722.17	84205.15	57423.18	0	0	0.56	0
SugarAlc2 (xylitol)	Sugar alcohol	NEG	74720.98	78397.69	50082.48	141413	114233.7	205171.9	155367.4	193347.3	204551.7	370496	619713.4	437741.7	8719.6	4294.57	11347.29	19721.83	927.91	11600.25	134.08	1041.31	79061	0	0	104.95	0	0	67.83	0
SugarAlc3 (C5)	Sugar alcohol	NEG	81806.38	109071.9	95739.68	173393	153137.6	255692.2	190176	245299.3	250230.9	413054.2	684270.5	345202.7	9081.93	4759.45	12009.35	1386.45	1137.03	11679.32	204.81	1525.49	27261	0	0	62.66	300.56	0	459.57	0

Table B. Nitrogenous Compound Peak Areas Used to Generate Figure 5 (Lower)

compound	Cpd Group	Polarity	820_0_10A.mzML	820_0_10B.mzML	820_0_10C.mzML	925_0_10A.mzML	925_0_10B.mzML	925_0_10C.mzML	927p1_0_10A.mzML	927p1_0_10B.mzML	927p1_0_10C.mzML	1002p1_0_10A.mzML	1002p1_0_10B.mzML	1002p1_0_10C.mzML	820_10_20A.mzML	820_10_20B.mzML	820_10_20C.mzML	925_10_20A.mzML	925_10_20B.mzML	925_10_20C.mzML	927p1_10_20A.mzML	927p1_10_20B.mzML	927p1_10_20C.mzML	1002p1_10_20A.mzML	1002p1_10_20B.mzML	1002p1_10_20C.mzML	820_20_30A.mzML	820_20_30B.mzML	820_20_30C.mzML	925_20_30A.mzML
Glutamine	Amino acid	NEG	256986.7	236140.1	276704.1	766924.4	934637.8	922289.9	1152454	969095.6	676665.8	3191908	3630482	4163007	1890.62	2850.84	1979.02	6215.68	5231.21	11955.51	10502.4	6357.78	12554.96	42323.36	65631	23559.22	0	0	446.58	269.94
L-isoleucine	Amino acid	NEG	1065698	1010940	1355437	2170234	2713559	2619372	2835559	2184772	1411965	3760236	6797298	4532155	534546.5	446002.7	539796.7	548822.1	675709	582346.9	610659.6	692886.4	848460	11361618	16327701	12290005	126766.4	324857.3	313584.7	282308.1
L-leucine	Amino acid	NEG	463493.7	454665.6	530390	889646.4	1030397	1007882	1218004	801366.5	643977.7	1438071	2856185	1850712	281129.2	219907.9	291866.1	278235.3	320281.8	267382.8	268756.4	313029.4	344835.8	3177363	4248999	3294488	105602.1	242596.6	244857.2	214714.3
Phenylalanine	Amino acid	NEG	412827.1	338346.3	518693.4	602824.7	735879.7	767631.1	674069.5	570584.2	454102.6	1111768	2761198	2078940	118646	106792.4	109949.4	120606	136349.3	128473.1	124957.4	142012.7	160016	1761245	2736600	1755793	134927.4	73941.03	74440.8	70109.84
Proline	Amino acid	NEG	50715.72	62258.94	72492.7	76856.86	94634.01	80880.16	77205.62	96113.9	54725.8	167667.6	426478	338806.7	4403.81	1430.93	4839.88	5860.98	5333.52	6908.64	3560.26	6714.1	17900.59	99228.71	124629.3	62012.16	646.94	539.41	456.42	1122.73
Tryptophan	Amino acid	NEG	162290.4	137105.1	220089.8	85362.98	107517.5	130120.8	210615.2	57789.69	57650.45	66653.12	679180	622934.5	6002.96	2960.29	5789.69	4001.8	2483.69	2775.85	3292.57	4648.15	5208.6	30771.73	59186.57	35868.68	3816.95	1587.74	1846.33	1914.37
Tyrosine	Amino acid	NEG	391998.9	286287.9	453513.7	303435.8	373269.2	372680.8	349651	261697.3	202946.7	316179.1	550960.7	441333.1	51322.17	44130.14	46109.71	40875.82	45164.1	41564.61	43327.87	54954	70642.3	269101.8	495277.6	347228	11123.58	686.59	15155.52	10090.58
Valine	Amino acid	NEG	267855.1	257902.7	359844.4	555837.6	696772.9	683530.7	742379.3	455920.6	308893	923533.6	1690090	1203068	53339.16	36382.45	54279.01	54017.89	75356.78	63065.24	56491.62	70975.96	82778.74	1385477	1862600	1339416	32387.2	22706.26	26914.35	21563.39
(4-aminobutanoate)	Amino acid metabolism	POS	8256019	7568733	7451032	7599228	8167552	7886034	8942831	11443612	8616646	8311729	9157441	9780039	932732	790367.8	835688.8	840843.6	760532.8	830154	1204107	1232509	1129340	2780480	2944276	2304534	421946.9	621525.7	583956.6	568609
(N-acetyl-l-phenylalanine)	Amino acid metabolism	NEG	192248.9	93832.1	206515.3	58833.63	72990.84	70554.15	54002.9	45409.11	39653.45	51353.99	74922.54	49707.93	7280.2	1480.89	5069.09	1537.51	1197.68	223.51	3835.65	5339.95	5629.58	1731.74	14398.52	10914.38	2459.3	4231.45	2419.81	1461.68
(C6H11NO3)	Amino acid metabolism	POS	18816203	36168296	26217857	998485.6	1115914	982451	1184399	1355050	1246927	5289726	2650502	3347142	5967713	7130488	5785588	3797367	4759447	5086618	5466161	5805744	6708713	3956401	4361783	3116068	755759.2	637666.4	521013.8	1182312
Deoxycytidine	Nucleotide/Nucleoside	POS	306100.2	269298.6	321992.4	426588.7	462901.4	384356.8	391270.5	357342.8	490957.9	377537.2	454700.7	387054.6	76706.19	109770.4	78720.6	263254.5	235080.3	226003.2	259858.6	207388.5	324062.5	1192683	1585001	1490457	55848.14	41946.56	34377.62	39295.62
Adenine	Nucleotide/Nucleoside	POS	1937859	2065954	2106092	1145734	1433305	1223654	1678647	5252373	1909302	1680503	2467421	1311560	753600.4	637247.9	668733.8	448217.2	405462.8	491659	521225.4	678104.1	833003.9	1026723	1174900	1753539	470783.7	506792.8	388254.2	356460.8
Adenosine	Nucleotide/Nucleoside	POS	23522068	23548075	25306651	9301647	14063825	12034529	10540881	9463261	13023048	7006982	9075516	10138348	3344628	3796920	4032039	3241816	2372240	2475316	3763324	3892717	5202812	2051265	4219437	4920370	1712596	2115849	2160503	1566640
Cytidine	Nucleotide/Nucleoside	POS	55821.57	54689.44	49866.13	44048.31	58771.68	65334.19	42434.71	42283.05	27382.44	30488.6	32730.92	41028.79	3427.81	3496.76	2687.31	1079.75	1075.63	940.82	2106.39	1338.28	2726.3	24659.03	53889.58	32887.98	0	1.24	0	15.57
Cytosine	Nucleotide/Nucleoside	POS	1889872	1815935	1839395	2609376	2969945	2639137	2283752	2198443	2765677	2262662	2669963	2285875	480950.4	601739.2	458859.1	1410709	1309388	1312481	1449911	1133305	1726197	7063701	9635699	8698693	273145.4	233292.9	188311.9	214872.6
Deoxyadenosine	Nucleotide/Nucleoside	POS	2594664	2226124	2519824	3904701	5034666	5512384	5375586	5470707	5093414	3734275	3983129	5490158	2100672	2720827	2602866	1748139	1925729	1590934	4279438	3421407	3631272	6527287	10880639	6606230	1739903	2368701	2377769	1625923
Deoxyguanosine	Nucleotide/Nucleoside	POS	302935.1	402403.3	712685.7	468263.3	964566.1	757903.6	578457.7	759807.2	193309.2	204362.8	315255.3	415956.9	0	0	0	130703.5	101381.9	37485.4	11091.4	0	15062.6	1467526	2723958	1658595	0	0	0	0
Deoxyuridine	Nucleotide/Nucleoside	NEG	143824.5	92081.43	35702.39	792108.3	962175.8	754199.9	633472.6	1464075	650703.5	1328275	1242214	1306554	233786.8	124026.4	287518.6	446997.8	194394.8	153444.1	641129	610300.7	634544	7503483	19737944	17738874	175257.8	185193.5	181998.4	125087.9
Guanosine	Nucleotide/Nucleoside	POS	821813.3	750764.8	721668.2	825390.9	1582048	1146369	707341.6	676242.7	542560	354101.9	375743.8	405132.1	256605.2	287714.9	195421.5	147860.5	159675.6	182259.3	158743.7	166125.4	196506.6	289772.9	385907.9	490169.9	73178.21	54472.03	40815.36	24408.89
Hypoxanthine	Nucleotide/Nucleoside	POS	1433670	1670761	1529649	1189244	1448215	1575275	1953903	1961344	1583133	1022482	1208226	1361748	157972.3	157006.3	148662.4	166772.4	144024.8	154312	215029.1	167217.8	291022.9	3028094	4547046	3679704	64255.48	81634.63	74005.17	24784.9
Inosine	Nucleotide/Nucleoside	POS	857401	831553.9	838096.6	687724	882077.2	955491.6	1324433	728093.1	1112714	554390	602865.7	751938	64322.22	67667.54	65100.57	69559.7	56419.97	63719.71	88755.64	73677.89	119217.5	219809.4	385182.8	416388.6	22833.81	28551.3	26215.44	12641.96
Uracil	Nucleotide/Nucleoside	NEG	2399983	1776959	2123504	2420802	2722612	2383347	2051426	2644655	2103942	2270028	2287493	1824659	1001955	300116.9	1152104	1095511	1060115	622638.4	1104478	1124682	1317408	4179101	8610523	8578221	592650.7	500990.4	468064.8	419894.3
Uridine	Nucleotide/Nucleoside	POS	110684.8	155955.7	158851.2	185281.3	234770	255938.2	208277.1	235333.5	140844.6	171782.1	225547	241902.8	67286.87	78074.49	62503.69	76997.63	63466.95	78433	79785.12	65161.2	84266.79	246461.4	371472.3	256659.7	16451.29	21245.21	18105.83	18355.84
Ectoine	Osmolyte	POS	17563064	18183129	18350468	6322075	6009664	5349918	6198746	7275906	6569070	4803854	5256382	5355894	911136.3	716984.5	739140.2	859492.9	704064.1	762669	1024000	995871.7	1810558	971187.8	1538203	1029035	157670.9	297627	235565.6	278596.7
(C8H16N2O3)	Peptide	POS	1.15E+08	1.13E+08	1.11E+08	91299208	94839896	83444994	76665107	86341745	77086559	1.35E+08	1.41E+08	1.37E+08	2522025	2567671	2403519	2434526	2809778	3371078	3713374	4139035	4690717	18575885	21419322	17846589	194341.5	228243.4	234656.4	157341.3
(C8H16N2O3)2	Peptide	POS	8506910	8027396	8178379	4987676	5494206	5016664	5405736	5153573	4178034	7914390	8270442	7835557	288063.7	244772.3	316160	202609.4	188569.9	210228.5	263123.5	335070	312500.7	497845.4	703281.8	441861.4	67802.11	111435.7	148648.4	90563.76
Acetylcarnitine	Quaternary NH4	POS	54776278	48032160	48749179	51589753	56014105	52586687	51009658	53895656	45425914	73499063	78316919	79684122	866217.4	911183.3	862598.2	631403.8	663616.5	728581.4	1558734	1479279	1713105	3291461	4297053	2172099	25475.13	55488.41	63435.83	44713.17
Betaine	Quaternary NH4	POS	1.29E+08	1.21E+08	1.16E+08	93087885	96844281	92540328	1.18E+08	1.27E+08	1.21E+08	1.31E+08	1.3E+08	1.28E+08	43748289	44607094	41559400	45635064	47228108	53043594	58035824	54439151	67439596	72374525	83599331	61919601	12489303	14019025	12965765	11957289
Choline O-sulfate	Quaternary NH4	POS	52643028	52562581	55067101	56136516	58818122	55280532	64730311	68877311	63411791	66588540	75413618	72138386	16543880	16614060	16131445	12604862	13714350	14543063	21797095	20904694	22268343	35714761	39915338	33584101	1243253	1586042	1448615	1302859
L-Carnitine	Quaternary NH4	POS	1.47E+08	1.4E+08	1.43E+08	1.3E+08	1.36E+08	1.18E+08	1.63E+08	1.68E+08	1.69E+08	1.55E+08	1.71E+08	1.63E+08	6299558	7528610	6482421	5500939	5644106	6927428	18441482	20496895	22765664	54630827	58867815	43837956	198589	284855.3	337155.7	213810.9
(Sarcosine)	Amino acid metabolism	POS	976173.4	811330.5	971240.9	1919981	2001133	1776955	2039225	2224836	1588781	2778033	3553286	3570678	374363.6	358718.2	462453	323811.9	239016	238877.4	355623	495065.7	331721.9	1862491	2250658	1691010	142396.4	270279.4	349270.1	253895.2
Serine	Amino acid	POS	223949.4	174439.2	203844.8	329120.5	358469.7	349880.4	364974.9	386423.6	301415.2	609822.8	1085642	1181981	58515.76	50842.23	67147.78	77658.32	66820.15	80604.49	66498.87	89774.69	108060.6	194147.4	223521.1	181998.1	54194.6	55035.1	61038.14	51324.62
Asparagine	Amino acid	POS	1647673	1383277	1416045	1915103	2291776	1727419	3120096	2456477	3039435	5156481	9810153	7375926	93999.31	95411.25	81979.61	64309.68	84223.3	93009.26	137180.7	181251.8	239588.3	241618.5	244309.2	171929.8	1193.74	4143.81	1867.73	2661.16
(N-Acetyl lysine)	Amino acid	POS	1.13E+08	1.12E+08	1.1E+08	90780280	94241503	83186587	76217558	86073293	76306784	1.34E+08	1.39E+08	1.36E+08	2531209	2579191	2434549	2474424	2848634	3398654	3737513	4160480	4677290	18688683	22910665	17963653	184198.7	220410.4	230047.2	149992.5

Table B. Nitrogenous Compound Peak Areas Used to Generate Figure 5 (Lower) (Continued)

compound	Cpd Group	Polarity	925_20_30B.mzML	925_20_30C.mzML	927p1_20_30B.mzML	927p1_20_30C.mzML	1002p1_20_30A.mzML	1002p1_20_30B.mzML	1002p1_20_30C.mzML	820_30_40A.mzML	820_30_40B.mzML	820_30_40C.mzML	925_30_40A.mzML	925_30_40B.mzML	925_30_40C.mzML	927p1_30_40A.mzML	927p1_30_40B.mzML	927p1_30_40C.mzML	1002p1_30_40A.mzML	1002p1_30_40B.mzML	1002p1_30_40C.mzML	927p2_10_20A.mzML	927p2_10_20B.mzML	927p2_10_20C.mzML	1002p2_10_20A.mzML	1002p2_10_20B.mzML	1002p2_10_20C.mzML	control1.mzML	control2.mzML	control3.mzML	control4.mzML
Glutamine	Amino acid	NEG	252.06	463.45	781.94	322.01	1129.14	982.55	913.13	139.19	172.86	486.57	332.56	472.24	251.77	57	422.68	324.97	153.23	0	248.72	8498.22	7167.65	6111.35	15364.27	44485.47	13102.5	630.88	0	883.19	247.28
L-isoleucine	Amino acid	NEG	238672.1	238393.7	341833.7	162124.3	292453.8	258265.3	237159.6	263820.9	277453.8	238573.3	278701.5	279475.3	245632	241020.3	230310.5	162434.9	264920.7	144656.8	266150.2	615071.6	542621.6	601356.1	774414.6	982223.1	689483.1	286860.1	322907.6	407364	391654.9
L-leucine	Amino acid	NEG	192962.5	178957	233185.3	197178.8	221341.8	203461.2	184206.7	208334.6	221810.3	187252.9	221417.9	187683.8	186238.7	176882.6	203783.4	124506.3	195709	93330.89	226564.8	293504.4	259727.9	284725	371392.3	442469.2	308883.2	285547	269357.7	388688.3	353382.5
Phenylalanine	Amino acid	NEG	58783.26	53691.83	77032.09	147103	77554.24	57912.1	92421.66	67453.42	67460.86	44239.51	119940	66913.71	50101.85	63796.75	47434.8	119905.9	57034.93	30608.9	69054.19	133402.5	114599.5	123151.3	126357.8	167049.1	123745.7	79940.91	64008.07	127127.7	84369.64
Proline	Amino acid	NEG	117.47	1151.31	1198.29	1745.82	2050.23	575.61	694.54	1161.34	382.73	1090.07	3952.18	508.98	78.49	636.16	567.13	984.11	623.48	328.76	1552.96	2982.6	2914.37	3622.65	9353.1	47637.15	5417.02	402.82	1083.89	1484.87	1264.4
Tryptophan	Amino acid	NEG	1357.63	1092.93	1834.69	3266.18	4635.54	2978.77	2388.35	2597.45	1461.43	1182.62	3695.45	2077.27	733.53	2144.07	1127.16	2906.19	1959.69	881.55	1877.32	4172.72	2159.36	5752.71	5199.11	125482.4	4450.76	2476.99	2386.01	3757.27	3460.99
Tyrosine	Amino acid	NEG	7772.21	8780.89	17228.94	11357.58	12883.28	11610.59	7705.37	9348.1	11911.12	6266.04	14391.88	7464.78	4130.59	6686.91	7853.18	3823.9	3983.74	885.88	9220.05	58200.66	49980.8	48086.11	48162.44	223737.7	41357.36	2417.94	4881.77	3761.38	5259.67
Valine	Amino acid	NEG	14136.48	15794.82	30501.75	38399.64	29371.51	20094.36	30451.71	23695.12	18649.31	15429.18	37268.39	23077.46	13663.78	18839.26	12645.43	28861.16	14676.74	6225.85	24476.37	55698.01	44239.51	50875.48	95603.47	309746.5	80905.86	26050.25	20655.28	35040.51	31447.59
(4-aminobutanoate)	Amino acid metabolism	POS	470369.8	445998.3	584417.2	440835.9	539422.7	468678	482197.2	450305.1	653737.1	494434.8	478195.8	462198.1	430575.2	503399.4	442103.9	406314.8	460676.3	422456.7	480851.2	1118794	1074042	1118105	987349.5	1845440	803573.3	342407.4	344134.5	418866	327523.2
(N-acetyl-l-phenylalanine)	Amino acid metabolism	NEG	949.13	378.85	4050.61	4421.69	6028.27	7074.91	279.32	2634.07	3030.83	2485.37	2128.49	1472.22	1456.62	1511.39	2454.46	2428.6	2742.75	287.76	3992.25	3545.83	2372.8	5454.05	10364.49	2936.95	4484.66	2089.03	3224.61	2932.36	3045.93
(C6H11NO3)	Amino acid metabolism	POS	1366113	1141615	1030173	833319	251494.7	254431.9	224342.4	492372.6	447922.7	450837.6	652866.5	484798	471502.5	255960.1	316205.1	293999.2	202628.8	180000.6	187012.2	3826944	4568359	5441102	3273949	2647246	1874634	123505.9	130332.8	117563.8	133765.9
Deoxycytidine	Nucleotide/Nucleoside	POS	44460.11	43716.94	96515.59	104821	34060.71	29761.24	29339.29	17411.81	14540.15	18792.38	22212.93	17892.6	19775.61	29916.76	27714.72	17865.89	3060.94	2632.75	3861.42	268186.5	272636.3	251081.5	137976.8	93506.5	100880.3	27.95	289.95	1062.12	254.23
Adenine	Nucleotide/Nucleoside	POS	420498.7	389836.2	408782.8	370969.1	250496.8	274493.1	229733.9	275743.6	275495.8	256735.1	425027.9	221164.4	242796.8	253386.7	196028.6	182650.3	127958	163949.6	179501.4	495797.8	495491.1	693221.5	326093.4	188195.6	207705.8	83974.88	101418.3	74517.11	84454.48
Adenosine	Nucleotide/Nucleoside	POS	1898802	1735792	2406863	2146130	758205.5	688057.2	580149.2	1392488	1393056	1529870	887634.8	1175170	1214732	972660.6	1325832	811744.5	195142	160270.7	161645.6	3594373	4449613	3847121	1667146	1107579	1135684	1171.02	2595.11	1982.58	1167.69
Cytidine	Nucleotide/Nucleoside	POS	94.94	53.78	78.44	0	94.34	85.76	0	104.07	0	0	52.81	0	0	0	0	0	0	0	27.65	1247.76	2601.15	1196.03	795.2	324.8	1153.11	0	0	275.21	0
Cytosine	Nucleotide/Nucleoside	POS	246133.3	253031.7	525532.7	533503.2	160095.6	177279.5	177361.5	94758.88	97717.25	113134.4	134024.6	113908.1	123898.3	184476.1	151320.6	101684.2	27510.06	26611.94	30781.33	1405924	1495258	1344549	719828.1	476630.4	530795.6	5159.32	9104.51	8710.9	6249.5
Deoxyadenosine	Nucleotide/Nucleoside	POS	1625161	1715209	4329835	3600526	752059.1	735990	707399.3	1456455	1622954	1925740	985282.7	1765198	1590653	2049371	2521032	1718271	184658.3	194455.4	111326.9	4464161	5008313	4527600	1106783	435930.2	617183.8	2787.58	1299.96	1358.08	2505.61
Deoxyguanosine	Nucleotide/Nucleoside	POS	0	0	0	0	0	0	0	0	0	0	0	0	0	0	0	0	0	0	0	156.9	10769	6705.1	0	0	0	0	0	0	0
Deoxyuridine	Nucleotide/Nucleoside	NEG	105043.8	49921.42	449115.3	356447.5	192775	183070.5	25630.27	118454.8	86776.31	98572.12	62857.82	59021.42	52305.14	97857.22	121263.7	114907	33167.62	7852.84	34406.22	423315.3	329605	713706.2	449128.2	150916	287406	1925.96	1594.85	1509.14	1979.68
Guanosine	Nucleotide/Nucleoside	POS	29301.71	27460.71	62206.41	61955.03	12143.95	21414.62	17104.71	32283.96	25433.43	23971.29	17136.44	11922.97	12997.36	16689.66	13651.42	19510.09	1329.5	1494.27	5635.11	139143.5	158819.7	171841.3	56527.88	37309.43	72724.54	3673.09	8745.09	479.24	5783.35
Hypoxanthine	Nucleotide/Nucleoside	POS	49018.94	16914.31	114816.1	76931.87	25521.61	25841.65	22318.43	55977.4	56599.19	42596.83	33257.64	23053.53	22931.04	84636.59	40412.6	21332.31	5767.67	2189.03	1847.56	161838.1	172249.3	214230.7	106733	44941.9	62620.31	31.13	316.26	196.57	0

Inosine	Nucleotide/Nucleoside	POS	23378.08	15054.47	42364.8	25845.67	10922.45	10881.62	10518.74	30945.31	30209.9	17796.86	9161.84	7090.76	7520.01	23650.9	12647.82	8180.14	531.83	972.63	725.97	59511.93	67861.88	64417.56	43500.27	21049.14	32462.19	0	5021.15	0	29.35
Uracil	Nucleotide/Nucleoside	NEG	383185.7	221893	793999	902187.4	485162.7	448530.4	72923.3	294063.4	197389.9	291576.7	226425.8	199633.7	158482.3	237455.1	236690.9	230501.3	147026.5	41203.26	155030.9	972617	886035.4	1219827	1198663	575252.1	938049	6396.61	2432.83	5730.69	6117.75
Uridine	Nucleotide/Nucleoside	POS	20285.57	18149.59	33246.27	30518.66	5723.24	8217.78	5000.45	5926.71	6286.3	5988.87	3686.25	7483.68	5157.48	6510.15	5974.27	3907.18	0.01	118.82	7.62	80063.18	94019.96	103809.5	54918.71	25857.3	37947.01	0	0	0	0
Ectoine	Osmolyte	POS	264784.2	256857.9	248276.2	233206.8	312157.2	251620.1	240003.9	214713.6	248738.6	185597.2	237278.4	198424	244251.6	176480.9	192952.9	161659.5	247739.7	222092.7	151928.2	458931.2	520549.5	653291.3	775975	797177.6	472233	180066.1	232691.7	253949.9	189669.2
(C8H16N2O3)	Peptide	POS	151576.1	128902.3	240971.9	289237.9	192417.6	182483.9	204266.3	93209.37	101999.7	74402.7	163053.5	87145.68	95641.71	73079.1	70031.32	76097.26	57054.67	58388.87	79255.75	1883822	2178719	2121215	2693786	4950854	2736149	50618.03	33499.48	331428.7	49526.26
(C8H16N2O3)2	Peptide	POS	82703.44	59596.21	95654.67	98844.77	101345.8	64280.97	63622.99	93960.77	118694.9	70086.76	100551	69643.81	67528.23	64005.35	74452.74	67841.2	61498.69	58124.68	65571.27	240568.5	226873.6	207431.7	241963	681061.8	200431.8	50014.93	44591.15	202050.6	46386.52
Acetylcarnitine	Quaternary NH4	POS	53488.37	30247.55	61352.58	44545.33	132824	116427.3	130742.8	38646.23	39062.46	35175.53	28845.33	30432.53	30613.96	34336.32	39073.28	12870.53	38512.51	29934.49	10376.34	1442478	1837451	1660050	2127274	3165266	2182013	24798.7	9331.3	116070.6	11120.42
Betaine	Quaternary NH4	POS	13728997	12596797	15136764	13981470	14696930	13462987	13548108	10726439	12139518	11490009	12371869	12121046	12216783	11421665	12052738	11024367	10773151	12123819	11522416	35788439	40603449	48661555	49832039	44145727	41187825	19122881	18823573	17422929	18850319
Choline O-sulfate	Quaternary NH4	POS	1733423	1379850	3399784	2335394	3251880	4102807	3525631	420924.5	444495.3	487036.3	889389.3	772939.2	964499.1	837567.6	748822.5	451233.6	576837.5	763858.6	663900.8	14525817	17494934	20022384	22754298	22432681	18901704	86.94	10.79	304.43	220.59
L-Carnitine	Quaternary NH4	POS	176308.6	175397.7	316178.8	359203.4	886744.3	696221.4	944005.2	173174.2	154775.1	103640.5	299318.7	126717.4	119317.9	122611.1	123623.5	110190.8	127415.4	112846	112904.1	20531543	22943837	26257846	11869665	21714574	10118362	92021.55	67355.6	488751.2	79015.82
(Sarcosine)	Amino acid metabolism	POS	196160.1	126382.4	255192.6	188802.8	375047.5	175331.2	175611.8	282283.4	300842	189131.8	293972.1	158577.5	150667.3	165198.9	187477.5	126244.6	197532	135374.2	214525.6	502256.2	418773.5	487769	657595.6	1356170	519882.7	106198.1	99955.57	165875.7	104185.9
Serine	Amino acid	POS	48151.92	46072.13	46199.22	58445.64	50316.84	36572.07	37205.82	30507.11	34688.96	29745.24	147304.3	39108.16	33343.71	28708.58	33970.77	34804.67	22981.61	15862.82	96903.2	78699.63	71088.18	88784.73	88480.71	150229.3	76849.47	15857.56	15722.25	25183.37	14587.82
Asparagine	Amino acid	POS	1570.63	1559.38	4944.49	4823.55	6959.77	4197.35	7937.43	1835.82	998.27	449.22	15862.93	2268.42	315.76	1004.31	538.64	313.32	428.89	184.98	3940	175871.4	177577	150381.2	140037.4	3186439	159655.7	632.11	1061.41	1050.26	832.55
(N-Acetyl lysine)	Amino acid	POS	145737.4	123371.2	246928.5	277019.6	206955	177509.1	201780.2	85074.45	88813.2	67662.9	149894.7	79971.78	88023.01	67700.46	61965.35	67630.7	50733.57	51078.3	72058.98	1883759	2199165	2155243	2708090	4990170	2757981	47983.26	29820.2	316230	45325.19
