# Supplementary material for: Proteogenomic analyses indicate bacterial methylotrophy and archaeal heterotrophy are prevalent below the grass root zone
Source: PeerJ. 2016 Nov 8;4:e2687. doi: 10.7717/peerj.2687 (PMC5103831; doi:10.7717/peerj.2687)
Supplement: Figure S2 — (A) Bacterial and archaeal16S rRNA phylogenetic tree. (B) Bacterial and archaeal rpS3 phylogenetic tree. All rpS3 proteins or 16S rRNA genes from every sample (colored branches) and the references (black branches, chosen based on closest hits to the sample sequences and representatives of in the NCBI database) were aligned and a phylogenetic tree was constructed using RAxML and visualized in Geneious (version 7.0 by Biomatters). [file peerj-04-2687-s002.pdf]

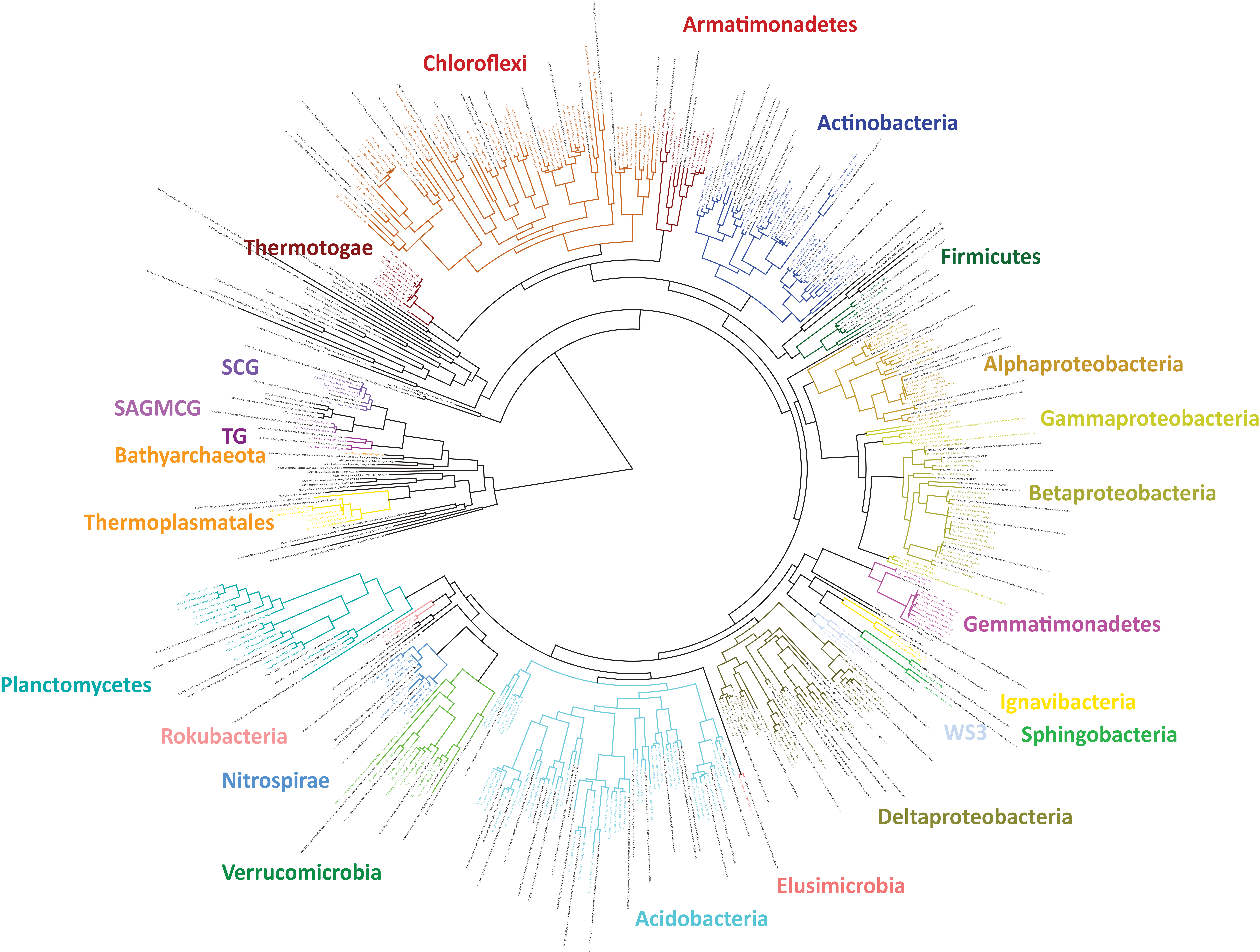
Armatimonadetes
Chloroflexi
Actinobacteria
Thermotogae
Firmicutes
SCG
Alphaproteobacteria
SAGMCG
Gammaproteobacteria
TG
Bathyarchaeota
Betaproteobacteria
Thermoplasmatales
Gemmatimonadetes
Planctomycetes
Ignavibacteria
WS3
Sphingobacteria
Rokubacteria
Nitrospirae
Deltaproteobacteria
Verrucomicrobia
Elusimicrobia
Acidobacteria

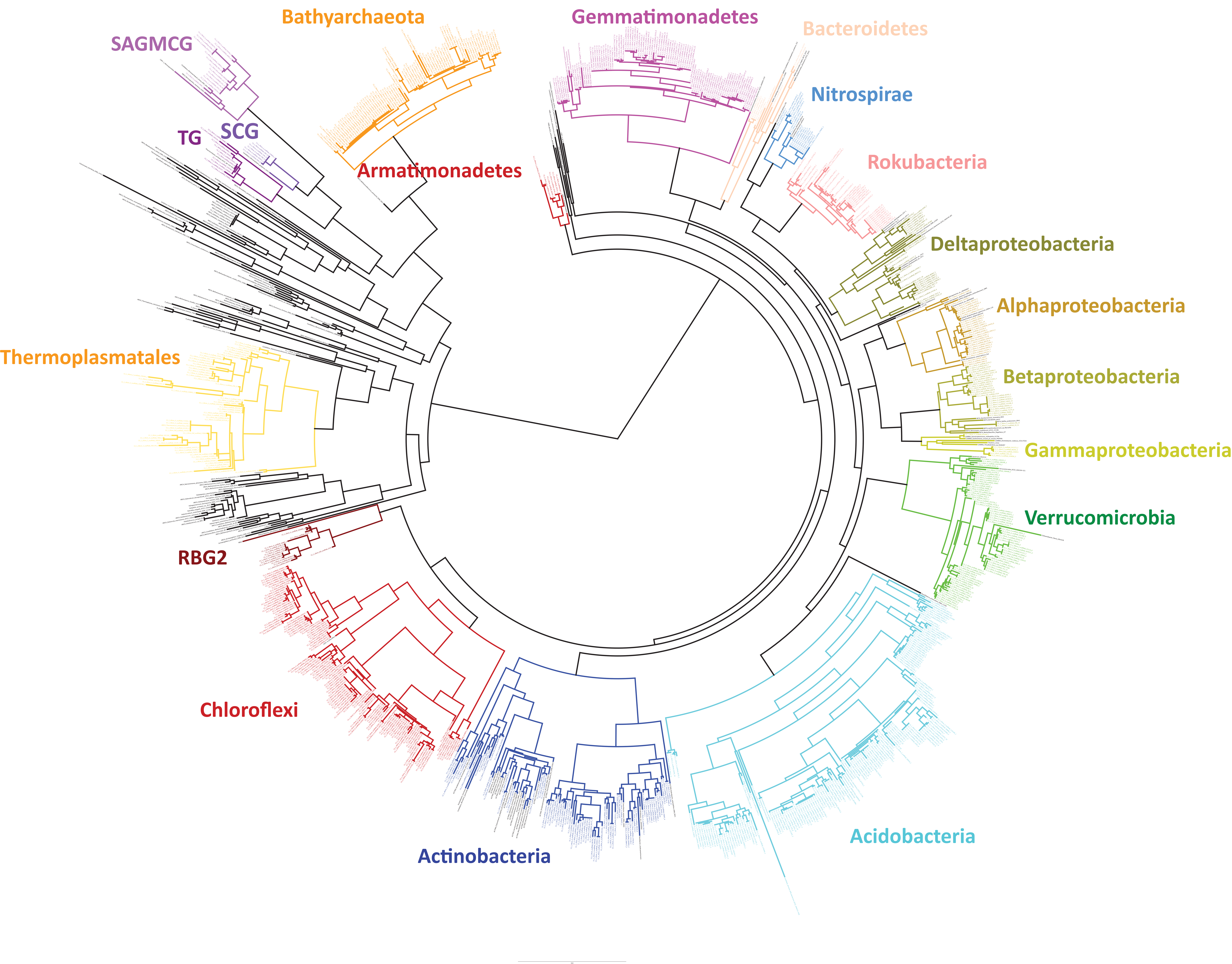
SAGMCG
Bathyarchaeota
Gemmatimonadetes
Bacteroidetes
Nitrospirae
TG
SCG
Armatimonadetes
Rokubacteria
Deltaproteobacteria
Alphaproteobacteria
Thermoplasmatales
Betaproteobacteria
Gammaproteobacteria
Verrucomicrobia
RBG2
Chloroflexi
Actinobacteria
Acidobacteria
